# Suppression of the *Escherichia coli dnaA46* mutation by changes in the activities of the pyruvate-acetate node links DNA replication regulation to central carbon metabolism

**DOI:** 10.1371/journal.pone.0176050

**Published:** 2017-04-27

**Authors:** Joanna Tymecka-Mulik, Lidia Boss, Monika Maciąg-Dorszyńska, João F. Matias Rodrigues, Lidia Gaffke, Anna Wosinski, Grzegorz M. Cech, Agnieszka Szalewska-Pałasz, Grzegorz Węgrzyn, Monika Glinkowska

**Affiliations:** 1 Department of Molecular Biology, University of Gdańsk, Gdańsk, Poland; 2 Department of Bacterial Molecular Genetics, University of Gdańsk, Gdańsk, Poland; 3 Institute of Molecular Life Sciences, University of Zurich, Zurich, Switzerland; Saint Louis University, UNITED STATES

## Abstract

To ensure faithful transmission of genetic material to progeny cells, DNA replication is tightly regulated, mainly at the initiation step. *Escherichia coli* cells regulate the frequency of initiation according to growth conditions. Results of the classical, as well as the latest studies, suggest that the DNA replication in *E*. *coli* starts at a predefined, constant cell volume per chromosome but the mechanisms coordinating DNA replication with cell growth are still not fully understood. Results of recent investigations have revealed a role of metabolic pathway proteins in the control of cell division and a direct link between metabolism and DNA replication has also been suggested both in *Bacillus subtilis* and *E*. *coli* cells. In this work we show that defects in the acetate overflow pathway suppress the temperature-sensitivity of a defective replication initiator–DnaA under acetogenic growth conditions. Transcriptomic and metabolic analyses imply that this suppression is correlated with pyruvate accumulation, resulting from alterations in the pyruvate dehydrogenase (PDH) activity. Consequently, deletion of genes encoding the pyruvate dehydrogenase subunits likewise resulted in suppression of the thermal-sensitive growth of the *dnaA46* strain. We propose that the suppressor effect may be directly related to the PDH complex activity, providing a link between an enzyme of the central carbon metabolism and DNA replication.

## Introduction

In their natural environment bacteria often come across drastic variations in growth conditions as well as competition with other species. During environmental alterations, bacterial cells need to constantly coordinate the chromosomal DNA replication and cell division with cell growth, to ensure faithful transmission of the genetic material to progeny cells while keeping the cell size homeostasis. Despite decades of research, the mechanisms coordinating these essential cellular functions remain poorly understood.

In *E*. *coli*, DNA replication is mainly regulated at the initiation level. At slow growth, cells contain only one replicating chromosome and the initiation of DNA replication is followed by complete chromosomal DNA synthesis and cell division in the same cell generation. However, during fast growth in rich media, cells attain bigger sizes, where the stable DNA/mass ratio is maintained due to a new replication round beginning before the preceding one finishes [1]. Hence, at fast growth rates, cells contain multiple replicating chromosomes although still only one replication initiation occurs per cell cycle, taking place in the mother or the grandmother cell generation. Results of early research on bacterial physiology, as well as the latest works employing live single molecule imaging suggest that replication is initiated at a constant, pre-defined cell volume per chromosome (origin), however the mechanisms coupling the cell growth with DNA replication are still not fully understood [2–5]. Several hypotheses were proposed to explain the mechanism of this coupling, where the most widely accepted one assumes that a threshold amount of an active initiator protein per origin must be attained to trigger initiation [6].

**Fig 1:**
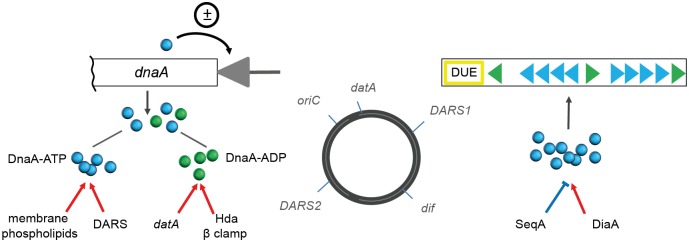
Mechanisms controlling DnaA abundance and activity in the *E*. *coli* cells. Left panel shows the factors stimulating ATP hydrolysis by DnaA or rejuvenation of DnaA-ATP. The *dnaA* gene and its promoter (gray triangle) are depicted. Right panel summarizes the mechanisms stimulating or blocking complex formation by DnaA-ATP at *oriC*. Stimulating interactions are marked by red arrows, inhibiting–by blue bars, ± sign depicts dual function of the DnaA-ATP protein as both, the repressor and activator of transcription. The *oriC* region contains three DnaA binding sequences of high affinity (green triangles), two arrays of low-affinity sites recognized by DnaA-ATP exclusively (blue triangles) and a DNA unwinding element (DUE) where duplex melting occurs. The middle circle reflects positions of the chromosomal regions involved in the regulation of DnaA activity (*datA* and two DARS) on the *E*. *coli* genome map and their relative distance to *oriC* and the *dif* chromosome dimer resolution locus, where chromosome copies become decatenated.

In *E*. *coli*, the replication initiator protein DnaA, when associated with ATP, forms an oligomeric complex of specific architecture facilitating the unwinding of DNA strands at the AT-rich region of *oriC* [7]–a prerequisite for subsequent helicase loading and assembly of the replication complexes. The ability of DnaA to form an oligomer competent in the DNA unwinding at *oriC* is tightly controlled by several mechanisms. One class of these mechanisms regulates the amount of DnaA-ATP in the cell and it may happen in two ways. Firstly, the ATPase activity of the DnaA protein is stimulated either by the Hda protein in cooperation with the β clamp of the DNA polymerase III or chromosomal *datA* region in the presence of the IHF nucleoid associated protein [8–11], reducing the amount of DnaA-ATP; secondly, an exchange of ADP to ATP by DnaA and hence—rejuvenation of the active form of the replication initiator protein—is facilitated by acidic phospholipids of the cell membrane, as well as by two specific regions of the chromosome called DARS [12, 13]. The second class of the DnaA-regulating mechanisms deals directly with the formation of the DnaA oligomer at *oriC*, with the DiaA protein stimulating this process and the SeqA protein hindering access of DnaA to the newly replicated origins and thus preventing premature reinitiation [14]. Moreover, DnaA-ATP (but not DnaA-ADP) can both effectively repress and activate transcription from the two *dnaA* promoters [15, 16]. Current picture of the cell cycle control involves coordinated action of the above mentioned mechanisms (summarized in Fig 1), resulting in fluctuations of the DnaA-ATP level during the cell cycle and reaching its peak at the time of DNA replication initiation. However, it is still not fully understood how fluctuations of DnaA-ATP are coupled to cell growth and whether reaching a certain DnaA-ATP threshold is a signal for replication initiation [17].

On the other hand, it is clear that cell growth is dependent on the overall metabolic status of the cell. Therefore, regulation of cell division and DNA replication has to be likewise coordinated with cellular metabolism. Recent data shows that a crosstalk exists between metabolic enzymes and the division apparatus. Namely, moonlighting metabolic pathway proteins were demonstrated to directly affect the activity of FtsZ in *E*. *coli*, *Bacillus subtilis* and *Caulobacter crescentus* [18–29]. There is also genetic evidence indicating direct influence of metabolism on the DNA replication process in *B*. *subtilis* [30–33]. Moreover, lysine 178 residing within the ATP-binding motif of DnaA and essential for nucleotide-binding function, has been shown to be acetylated in *E*. *coli* cells by the YfiQ acetyltransferase, in a growth phase-dependent manner (with the highest acetylation level during the stationary phase). This modification results in the inability of DnaA to form a complex at *oriC in vitro* [34]. It is possible that variable level of DnaA acetylation under different growth conditions could provide a direct link between cellular metabolism and control of the DNA replication initiation. Also, deletion of various genes encoding enzymes involved in the central carbon metabolism resulted in suppression of phenotypic manifestation of several replication proteins‘ defects in *E*. *coli* [32–33], however the mechanisms of suppression were not characterized.

Particularly interesting was the case of an effective suppression of the temperature-sensitive growth and other phenotypes of the *dnaA46* mutant strain, by the lack of either of the two genes of the acetate overflow pathway [32–33]. The DnaA46 protein variant contains A184V and H252Y substitutions, residing within the domain responsible for ATP binding. These changes confer a defect in nucleotide binding and thermolability in the initiation of *E*. *coli* DNA replication [35].

Pta-AckA pathway produces acetate from acetyl-CoA in two steps, with concomitant synthesis of an ATP molecule. It is utilized in *E*. *coli* cells during growth under anaerobic, but also fully aerobic conditions in media rich in glucose. In the latter case, it results in reduction in the flow of metabolites through the TCA cycle and electron transport chain and diverts a part of the substrate (glucose) to production of an incompletely oxidized product–acetate. Several, not mutually exclusive roles, have been proposed for this seemingly wasteful pathway. Namely, the reactions carried out by Pta-AckA could allow for fast growth by replenishing the free CoA-SH, a co-factor of 2-oxoglutarate dehydrogenase (KGDH), under conditions of high acetyl-CoA synthesis by pyruvate dehydrogenase [36]. Moreover, it was proposed that, due to repression of the metabolic flux through the TCA cycle, which occurs concomitantly with acetogenesis at higher growth rates, it allows for maintaining optimal NADH/NAD ratio. This prevents redox imbalance which results in formation of oxygen radicals, while keeping a necessary pool of the free NAD+. The flow through the acetate overflow pathway was proven to depend itself on a sufficiently high NADH/NAD ratio [37, 38]. In addition, its intermediate metabolite (acetyl-phosphate) functions in the post-translational protein modification: phosphorylation and acetylation [39–41]. Recently, it has been shown that diverting the flow of metabolites to a less efficient but also less costly pathway in terms of protein production (the TCA cycle and electron transport chain vs the acetate overflow pathway), may allow the cell to redirect the released fraction of the cellular protein content to translational machinery, and thus–to increase the biomass synthesis capacity of the cell, enabling fast growth [42]. Strains bearing the *pta* or *ackA* mutation display a reduced growth rate under acetogenic conditions and were also demonstrated to upregulate some stress response genes, in particular these responsible for acid resistance [43], however the reason for those changes is not clear.

The mechanism of *dnaA46* phenotypes suppression by deletion of one of the acetate overflow pathway genes is not known. Studying the cumulative effects of mutations in both the replication and metabolic genes, may bring an important insight into the alterations of the activity of replication proteins exerted by metabolites, metabolic enzymes or post-translational modifications. Characterizing these mechanisms will shed light on the nature of links between metabolism and replication and answer the question whether direct crosstalk between these processes could play a role in the cell cycle regulation.

## Materials and methods

### Strains and growth conditions

Strains with deletions of several genes (*pta*, *ackA*, *lpd*, *aceE*, *rpoS*, *gadX*, *yfiQ* and *cobB*) were previously described [44] and were obtained from the Yale CGSC collection. The CF1693 strain (*relA*::*kan*, *spoT*::*cm*) was used as a donor for construction of the *relA* and *spoT* knock-out mutants [45], and *dnaA46 tnaA*::*tet* strain [46] was used for construction of *dnaA46* derivative of MG1655. All MG1655 derivatives [47] were constructed by P1 transduction; the presence of knock-outs was confirmed by PCR and sequencing.

The pTrc99-nox plasmid [37] was obtained from Prof. Mark A. Eiteman (University of Georgia, Athens, USA). The empty vector pTrc99 was constructed by digestion of pTrc99-nox with *Bam*HI and *Pst*I, creation of blunt ends with T4 and Klenow polymerase and religation. pTrc99-*fdh* was obtained similarly, except that a PCR fragment obtained using primers complementary to the *Candida boidinii fdh* gene, digested with *Bam*HI, was cloned into the *Bam*HI-*Pst*I sites of the pTrc99-nox plasmid. Protruding overhangs remaining after the *Pst*I digestion of pTrc-nox were removed by T4 polymerase prior to ligation with the DNA fragment containing *fdh*. Sequences of primers used for the *fdh* amplification from the *Candida boidinii* chromosomal DNA were as follows:

5’- TGTGGGATCCATGAAGATTGTCTTAGTTCTTTATG and 5’- CTATTTCTTATCGTGTTTACCGTAAGC.

Unless indicated otherwise, bacteria were grown in the LB (Lennox) medium at 30 (permissive) or 39 (restrictive) °C. The M63 minimal medium, containing glucose or glycerol was used as described previously [43]. Acetate (potassium or sodium salt) and pyruvate (sodium salt) were added to the LB medium at indicated amounts, and the medium was buffered with 50 mM TES-KOH pH 7.0.

### Growth conditions and mRNA isolation for RNAseq

The *Escherichia coli* MG1655 strain and its derivatives were grown at 30°C in LB medium. The overnight culture was diluted 1:1000 in a fresh LB medium and cells were grown with aeration until the OD_600_ of the culture reached 0.3. To each sample (10^9^ cells), ice-cold Ethanol/Phenol (5% Phenol) solution was immediately added (1:8 ratio of the stop solution: culture) to prevent mRNA degradation; the cells were then harvested by centrifugation and the pellets were frozen in liquid nitrogen. RNA was extracted with the RNeasy Mini kit (Qiagen) and treated with Turbo DNase (Life Technologies) according to the manufacturers’ instructions. Subsequent rRNA depletion was carried out with a Ribo-Zero kit (Illumina) and 0.5 g of the enriched mRNA of each sample was sent for RNA-seq analysis (Illumina HiSeq 2000).

### Gene expression analysis

Sequence reads were mapped using bowtie2 (version 2.2.3) to the *Escherichia coli MG1655* reference genome available from the NCBI refseq database (accession: NC_000913.3). Gene count tables were generated from the bowtie2 alignments for each strain and replicates. Finally, a differential expression analysis was performed on the gene counts using the EdgeR bioconductor package for the R statistics language. Statistically significant differences in expression of gene sets across strains were tested using the ROAST method available in the same package.

### Determination of pyruvate, 2-oxoglutarate and acetate level

Acetate and pyruvate concentration in bacteria was assessed using the FluoroSELECT^™^ Acetate Assay Kit (Sigma-Aldrich) and Pyruvate Assay Kit (Sigma-Aldrich), respectively. Briefly, an overnight culture of bacteria was diluted 100-times in a fresh LB medium, and cultivation was continued at 39°C, with shaking. Samples (0.5 ml) were withdrawn at OD_600_ = 0.3, chilled on ice and spun down. 450 μl of bacterial supernatant was discarded and the pellet was suspended in 200 μl of the Acetate Assay Buffer (acetate quantification procedure) or the Pyruvate Assay Buffer (pyruvate quantification procedure). Bacteria lysis was performed by sonication. The debris was spun down and 1:10 dilution were made in appropriate assay buffers. Fluorometric quantification of acetate and pyruvate was completed according to the manufacturer’s protocols. 2-oxoglutarate level was measured using cold methanol extraction [48] and the α-Ketoglutarate Assay Kit (Sigma).

### Determination of NADH/NAD ratio

NADH/NAD ratio was assessed using NAD/NADH quantitation kit (Sigma-Aldrich). Briefly, bacteria were cultivated at 30°C in LB medium to OD_600_ ~ 0.5, subsequently a sample conaining ~1.25∙10^10^ was added to 25 ml of cold methanol (-80°C) and centrifuged at 10000 g for 5 min at 0°C. Supernatant was removed; pellets were immediately frozen in liquid nitrogen and stored at -80°C no longer than 24h. Prior to measurement, pellets were resuspended in 250 μl of NADH/NAD extraction buffer, sonicated and centrifuged for 5 min at 15000 g. Samples were deproteinized by filtering through 10 kDa cut-off spin filters. 50 μl of samples were measured according to manufacturer’s instructions.

Measurement of NADH/NAD ratio in the MG1655 strain containing plasmids overexpressing formate dehydrogenase (*fdh1*) or NADH oxidase (*nox*) genes was performed in a similar way, but at OD_600_ 0.1–0.15 bacteria were induced with 1 mM IPTG and samples were taken at OD_600_ ~ 0.7.

## Results

### Suppression of the *dnaA46* growth defect by the deletion of *pta-ackA* pathway occurs during fast growth under acetogenic conditions

**Fig 2:**
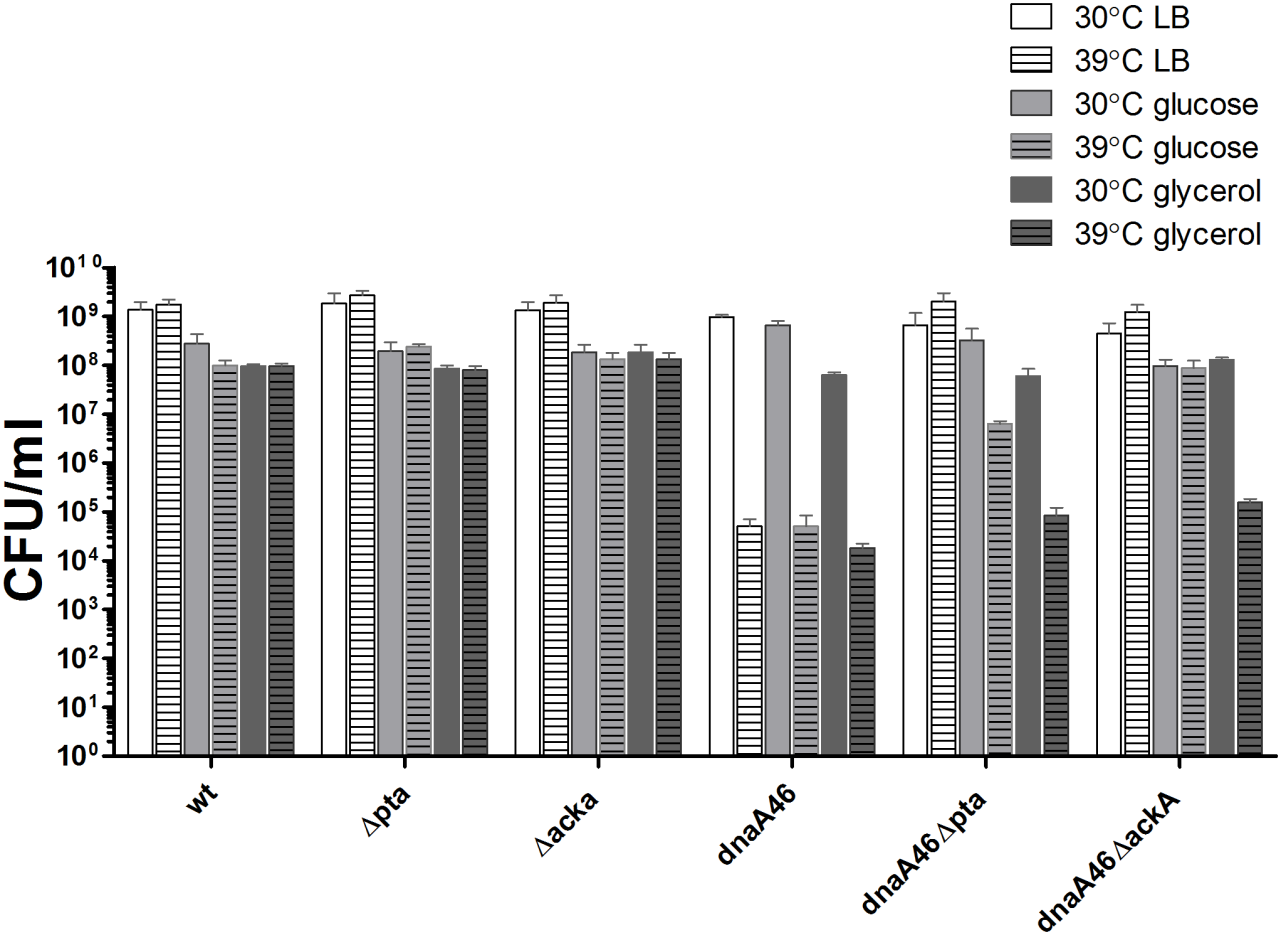
Suppression of *dnaA46* thermal sensitivity by mutations in the *pta-ackA* pathway depends on the carbon source. Bacteria were grown on plates containing the indicated carbon sources (as described in Materials and methods) at 30 and 39°C and growth was estimated in CFU. Data represents mean ± SEM of three independent experiments.

In order to shed light on the mechanism of suppression of the *dnaA46* phenotypes by the dysfunction of the acetate overflow pathway, we asked whether it depends on the lack of the Pta and AckA proteins *per se* or on metabolic changes occurring in the cells in these proteins’ absence. The growth of *pta* and *ackA* mutants is perturbed in comparison to their wild-type counterpart in acetogenic media supporting fast growth but not in media containing glycerol as a sole carbon source (Fig A in S1 File) [49]. Therefore, we speculated that the suppression of *dnaA46* phenotype caused by the deletion of *pta* and *ackA* genes may be condition-dependent and relies on the changes in cellular physiology arising during growth of the acetate pathway mutants on an acetogenic medium. In other words, we assumed that the suppression occurs under conditions where the metabolism of *pta* and *ackA* mutants differs significantly from the wild-type strain. To test this hypothesis, we compared the ability of *dnaA46 Δpta* and *dnaA46 ΔackA* strains to form colonies at 39°C, during growth on minimal medium containing glycerol, on a glucose minimal medium and LB (Fig 2). The growth of *dnaA46 Δpta* mutant at the restrictive temperature was already reduced in the glucose-minimal medium in comparison to that observed on LB, whereas suppression of the *dnaA46* temperature-sensitivity was abolished for both double mutants when utilizing glycerol as a sole carbon source (Fig 2). Dependence of suppression on the medium composition again strongly indicated that the function of DnaA46 protein at an elevated temperature is restored due to metabolic alterations in the Δ*pta* and Δ*ackA* mutant cells growing in acetogenic media supporting a fast growth rate. Moreover, this result further confirmed that the suppression, observed in the LB medium, is not a simple consequence of the slower growth rate of the mutants, since if this was the case, the observed suppression should be strongest in media supporting slow growth, i.e. in glycerol. In addition, we also observed growth of the *dnaA46 Δpta ΔackA* triple mutant at the restrictive conditions in LB medium, confirming that the phenotypic suppression is a result of alterations arising in response to a malfunction in the acetate overflow pathway (Fig C in S1 File).

**Fig 3:**
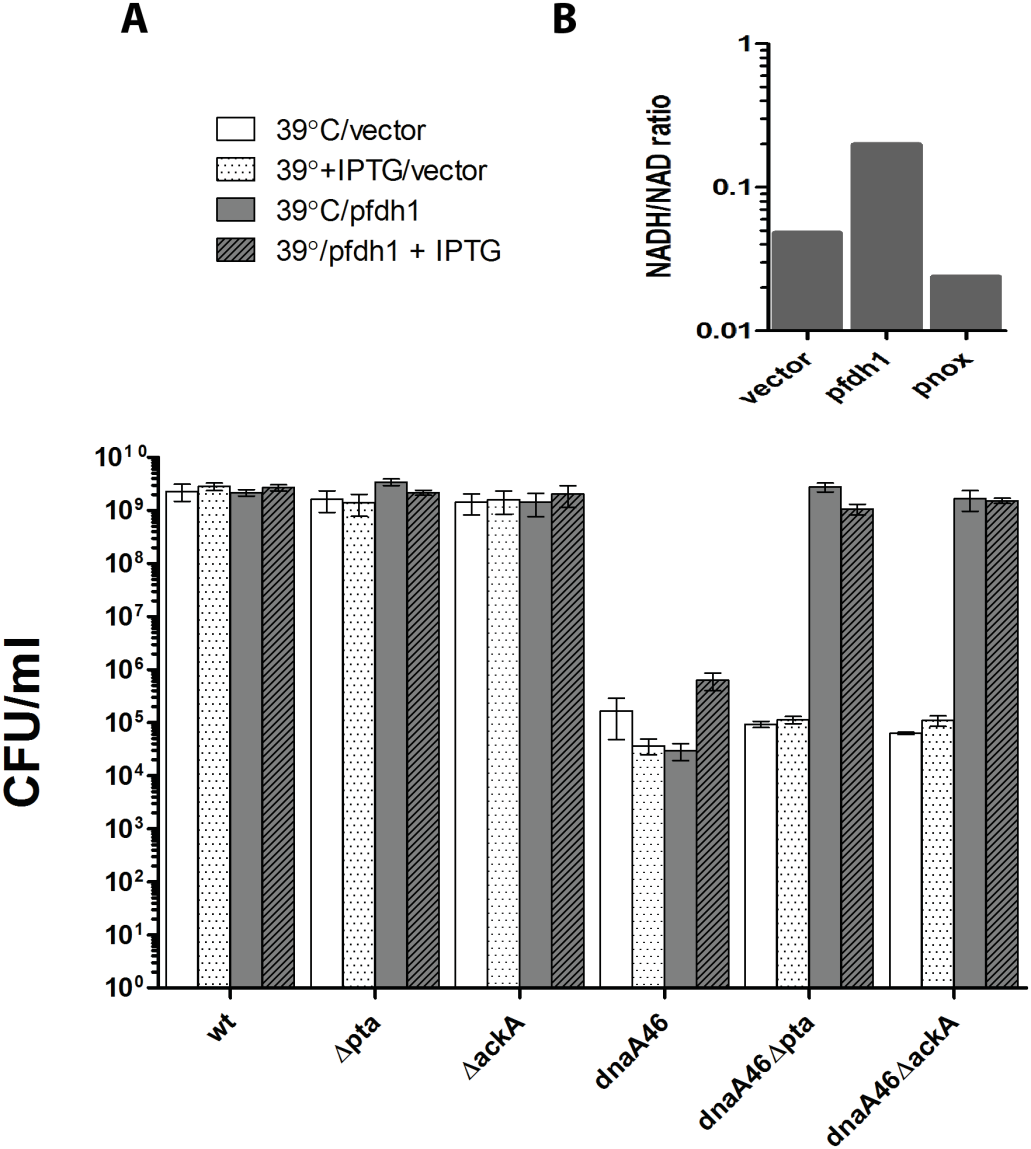
The effect of suppression of *dnaA46* replication defect by mutations in the acetate overflow pathway can be modulated by manipulating NADH/NAD+ ratio. Bacteria were grown on LB plates containing 100 mM sodium formate and 1 mM IPTG (where indicated). Growth was estimated in CFU. Data represents mean ± SEM of three independent experiments.

In order to confirm that the suppression of the *dnaA46* phenotype depends on the activity of the *pta-ackA* pathway under conditions of fast growth in an acetogenic medium, we made an attempt to manipulate utilization of this pathway in the *dnaA46 Δpta* and *dnaA46 ΔackA* cells growing in the LB medium. Flux through the acetate overflow pathway was shown to be correlated with the redox ratio. Namely, a switch from respiratory to respirofermentive metabolism occurs at a certain threshold value of NADH/NAD+ (0.06), whereas overexpression of the NADH oxidase, reducing this ratio, decreases acetate formation [37]. Therefore, we introduced enzymes, which were previously shown to increase or decrease the redox potential in *E*. *coli* cells, by overproducing *Streptococcus pneumoniae* water-forming NADH oxidase (NOX) or *Candida boidinii* NAD+ dependent formate dehydrogenase (FDH), respectively [37, 38]. Since overproduction of these proteins is not very efficient in *E*. *coli*, even when the gene is present on a multicopy plasmid (data not shown), and the above mentioned changes in the acetate excretion were measured for proteins produced from a plasmid-borne gene [37, 38], we attempted to use a system similar to the previously published ones. The *nox* and *fdh* genes were placed under an IPTG-inducible promoter in the pTrc99a-based plasmids. However, the empty vector alone severely reduced the suppressor effect of metabolic mutations with respect to *dnaA46*, most likely masking the effect of the NOX activity (Fig D1 in S1 File). Nevertheless, when FDH was overproduced, and thus the NADH/NAD+ ratio increased, the ability to grow at the restrictive temperature was restored to the *dnaA46 Δpta* and *dnaA46 ΔackA* strains bearing the respective plasmid (Fig 3). On the other hand, the presence of a plasmid expressing the *pncB* gene, resulting in the synthesis of additional NAD+ and thus leading to lowering of the intracellular redox ratio, was not able to restore the suppression (data not shown). The *pncB* gene codes for nicotinic acid phosphoribosyltransferase (NAPRTase) which catalyzes the formation of nicotinate mononucleotide, a direct precursor of NAD, from nicotinic acid. In accordance with these results, NADH/NAD ratio was increased in the cellular extracts of strains with deletions of either *pta* or *ackA* gene (Fig D2 in S1 File) and these strains had higher level of total NAD(H) amount (particularly the *pta* mutant), suggesting increased synthesis of the cofactor (data not shown).

The results presented in the above paragraph strongly suggest that the growth of *dnaA46 Δpta* and *dnaA46 ΔackA* double mutants at elevated temperatures results from metabolic alterations which occur under conditions supporting high growth rates, when the increase in the intracellular redox ratio is not accompanied by the activity of the acetate overflow pathway.

### Strains lacking a part of the acetate overflow pathway upregulate σ^S^ and ppGpp-dependent genes and accumulate intermediary metabolites during growth under acetogenic conditions

To gain insight into the possible cause of suppression, we compared transcription profiles of the *dnaA46* mutant and the strains bearing in addition suppressor deletions in *pta* or *ackA*. We did not observe significant differences in the RNA level of *dnaA* or its known regulators (*hda*, *diaA*, *dnaN*, *seqA*) between the *dnaA46* strain and its counterparts containing the suppressor mutations. However, the overall transcriptomic profiles of *Δpta* and *ΔackA* strains differed significantly from that of its wild-type counterpart (Fig B in S1 File).

**Fig 4:**
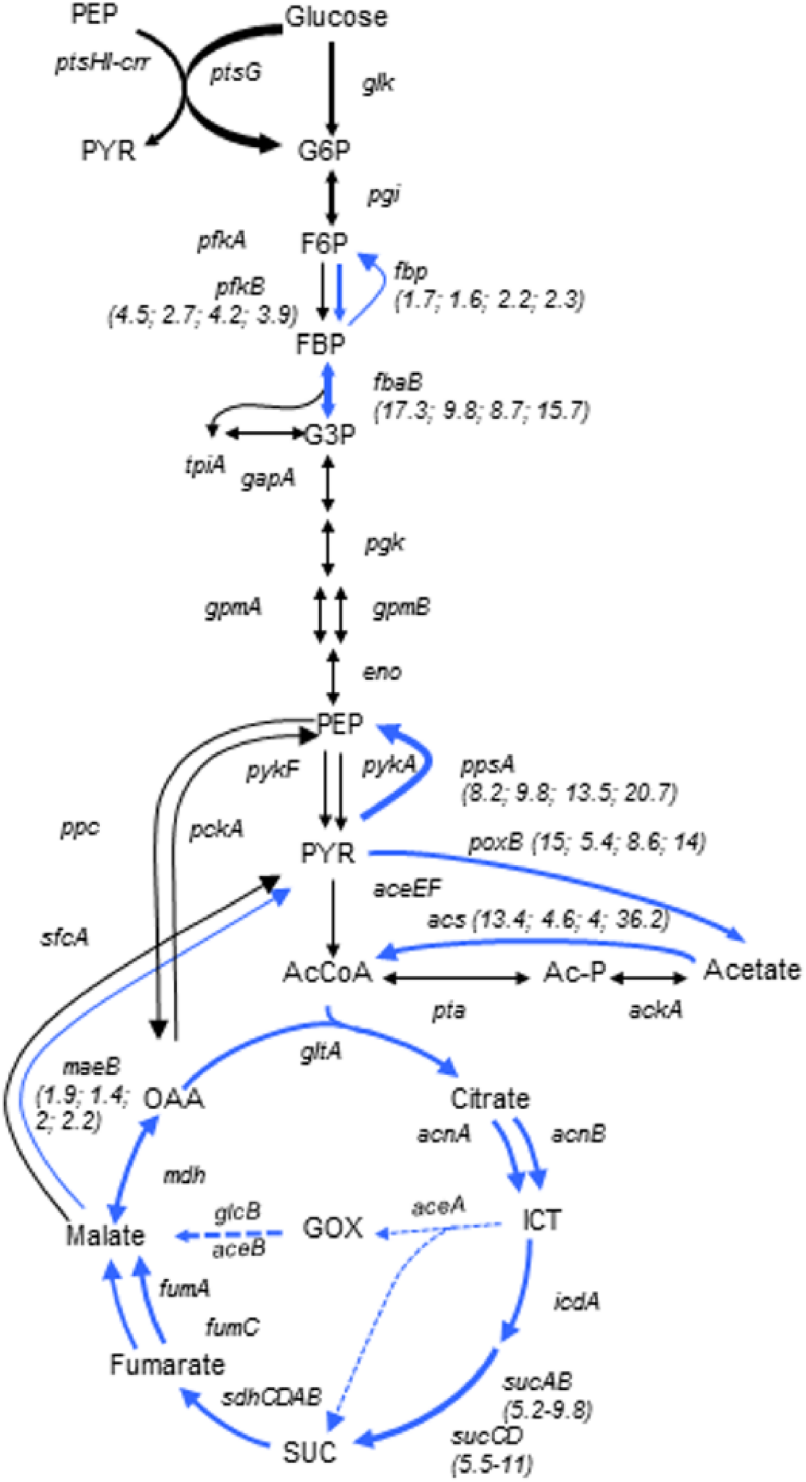
Alterations of CCM genes’ expression in the single and double metabolic mutants. Blue arrows indicate upregulation of transcription of the respective genes. Only the genes that were consistently upregulated at least 2-fold in all of the four strains bearing metabolic mutations (*ackA*, *pta*, *dnaA46ackA* and *dnaA46pta*) were marked by solid blue arrows. The arrows represent gene function and not reactions, therefore two arrows are present for the functions encoded by two separate genes which may be differentially regulated. The expression of glyoxylate bypass genes was increased more than 2-fold in both *ackA* strains, but not the *pta* mutants, although it was still enhanced in the latter strains in comparison to the *wt* (as marked by blue dotted lines). Relative change of RNA abundance is shown as fold change (values in brackets) for significantly upregulated genes. Consecutive numbers stand for the fold change in the *ackA*, *pta*, *dnaA46ackA* and *dnaA46pta* strain, respectively. All of the TCA cycle genes were upregulated by more than 2 fold in all four strains, the change of expression *of sucAB* and *sucCD* was most pronounced and the range of fold-change in all strains was indicated in brackets. Abbreviations: G6P –glucose 6-phosphate, F6P –fructose 6-phospate, FBP- fructose 1,6-bisphosphate, G3P –glyceraldehyde 3-phosphate, PEP–phosphoenolpyruvate, PYR- pyruvate, AcCoA–acetyl-CoA, ICT–isocitrate, SUC–succinate, OAA–oxaloacetate, GOX–glyoxylate. *pstHI*, *crr*, *ptsG*–PTS glucose transport system, *glk*—glucokinase, *pgi*–phosphoglucose isomerase, *pfkA* and *pfkB*– 6-phosphofructokinase, *fbp–*fructose-1,6-bisphosphatase, *fbaB*–fructose bisphosphate aldolase, *tpiA*–triosephosphate isomerase, *gapA*–glyceraldehyde 3-phosphate dehydrogenase, *pgk*–phosphoglycerate kinase, *gpmA* and *gpmB*–phosphoglycerate mutase, *eno*—enolase, *pykA* and *pykF*–pyruvate kinase, *ppsA*–phosphoenolopyruvate synthetase, *pckA*–phosphoenolopyruvate carboxykinase, *ppc*—phosphoenolopyruvate carboxylase, *poxB*–pyruvate oxidase, *aceEF–*pyruvate dehydrogenase, *acs*–acetyl-CoA synthetase, *sfcA* and *maeB–*malate dehydrogenase, *gltA*–citrate synthase, *acnA* and *acnB*–aconitate hydratase, *icdA*–isocitrate dehydrogenase, *sucAB*– 2-oxoglutarate dehydrogenase, *sucCD*–succinyl-CoA synthetase, *sdhCDAB*–succinate dehydrogenase, *fumA—*fumarase, *mdh*–malate dehydrogenase, *aceA*–isocitrate lyase, *glcB* and *aceB*–malate synthase. The scheme was adapted from reference 50.

Expression of the central carbon metabolism genes was altered in both the single and double mutants in a similar way, irrespective of which particular element of the acetate overflow pathway was inactivated. The mutant strains showed an elevated expression of genes encoding: the TCA cycle enzymes, acetyl-CoA synthetase (*acs*), pyruvate oxidase (*poxB*), gluconeogenetic enzymes *ppsA*, *maeB*, *fbaB* and *fbp* (Fig 4). On the other hand, the expression of many genes, coding for transport proteins for alternative carbon sources, was substantially elevated, for example: operons *mglBAC* (galactose), *gatYZ* (tagatose), *malEFG* and the *malK* and *lamB* genes (maltose) (Table B in S1 File). Overall, in the double and single overflow pathway mutants, activity of the genes of central metabolic pathways and transport proteins resembled that which was previously shown for acetate-grown *E*. *coli* cells [50].

Significantly, genes associated with a stress response were differentially expressed in all of the tested strains containing mutations in the overflow pathway genes. A large fraction of the genes (over 30%) belonging to the *rpoS* regulon was upregulated more than twofold in the *ΔackA* mutant. In the *Δpta* strain, the percentage of upregulated *rpoS*-dependent promoters was on the whole lower, however, expression of many general stress response genes was still higher in comparison to the wild-type strain, especially those connected with response to acidic and osmotic stresses as well as carbon and nitrogen starvation and acetate metabolism (Fig D in S1 File, Table A in S1 File). Our results corroborate findings of previous studies which pointed to an elevated level of some transcripts and proteins regulated by σ^S^ in the acetate overflow pathway mutants growing in the LB medium [43, 51].

**Fig 5:**
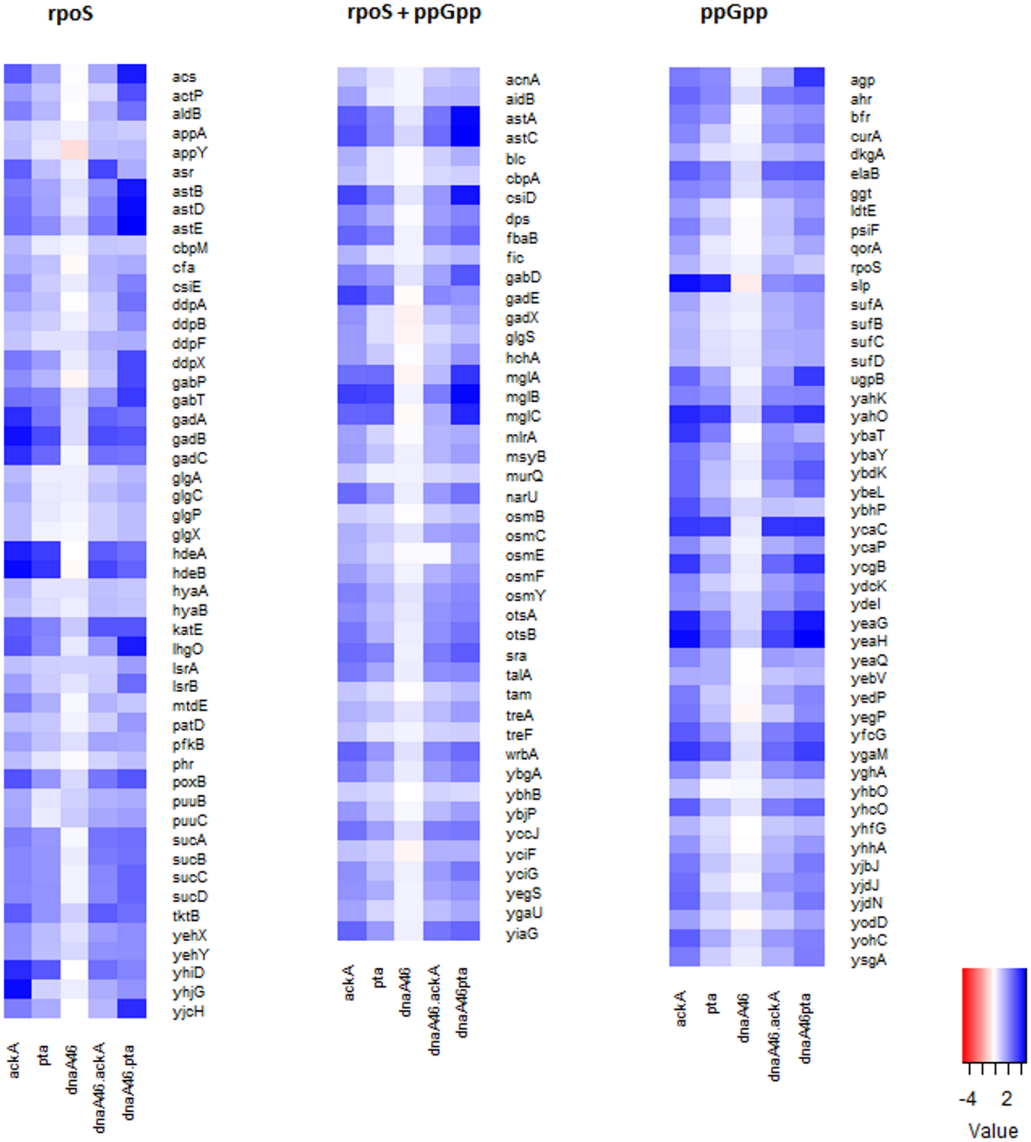
Expression of genes regulated by *rpoS* and ppGpp in the overflow pathway mutants. Gene expression was estimated in the exponential phase by RNA-seq. Heat map contains the genes whose expression in the metabolic mutants was changed by at least 2-fold with p-value < 0.05. RpoS and ppGpp-regulated genes were chosen based on results of whole transcriptome studies of an isoleucine limitation response [52].

Genes of the *rpoS* regulon are strongly induced under a variety of stress conditions including starvation, as a result of an increase in the level of ppGpp. This alarmone is produced by RelA or SpoT proteins in response to amino acid starvation or limitation of several other compounds (fatty acids, carbon, iron, nitrogen), respectively. Therefore, we asked, whether the observed upregulation of the stress response genes could be associated with ppGpp-dependent regulation. We analyzed our transcriptomic data using a set of *rpoS*-dependent genes activated upon amino-acid starvation which had been previously defined in experiments with wild-type and *rpoS* mutant strains growing under isoleucine limitation conditions [52]. Comparison of this set to the pool of genes upregulated in the acetate overflow pathway mutants revealed that the majority of genes sensitive jointly to *rpoS* and ppGpp (rpoS+ppGpp) are highly expressed in the *ΔackA* cells (>70%) and a lower but substantial fraction of these genes, was also activated in the *Δpta* mutant (Table A in S1 File). Thus, the transcription profile of the acetate overflow pathway mutants grown in a rich medium, particularly *ΔackA*, is akin to the one observed in cells under amino acid starvation conditions (Fig 5, Table A in S1 File, Fig D in S1 File).

This expression pattern was preserved in the *dnaA46 ΔackA* double mutant, where abundance of most of the *rpoS* and *rpoS*+ppGpp dependent genes (genes differentially expressed in the presence of high ppGpp levels and absence of *rpoS*) was still significantly increased in this strain, although, in many cases, to a lesser extent than in the single *ΔackA* mutant. On the other hand, expression of these genes increased in the *dnaA46 Δpta* mutant in comparison to the *Δpta* strain, making the transcription pattern of the former strain similar to that of *ΔackA*, and resulting in both double mutant profiles being more closely related than those of the single mutants. As expected, the expression patterns of cells containing the *dnaA46* allele and the wild-type ones were very similar, with only few differences (Fig 5).

**Fig 6:**
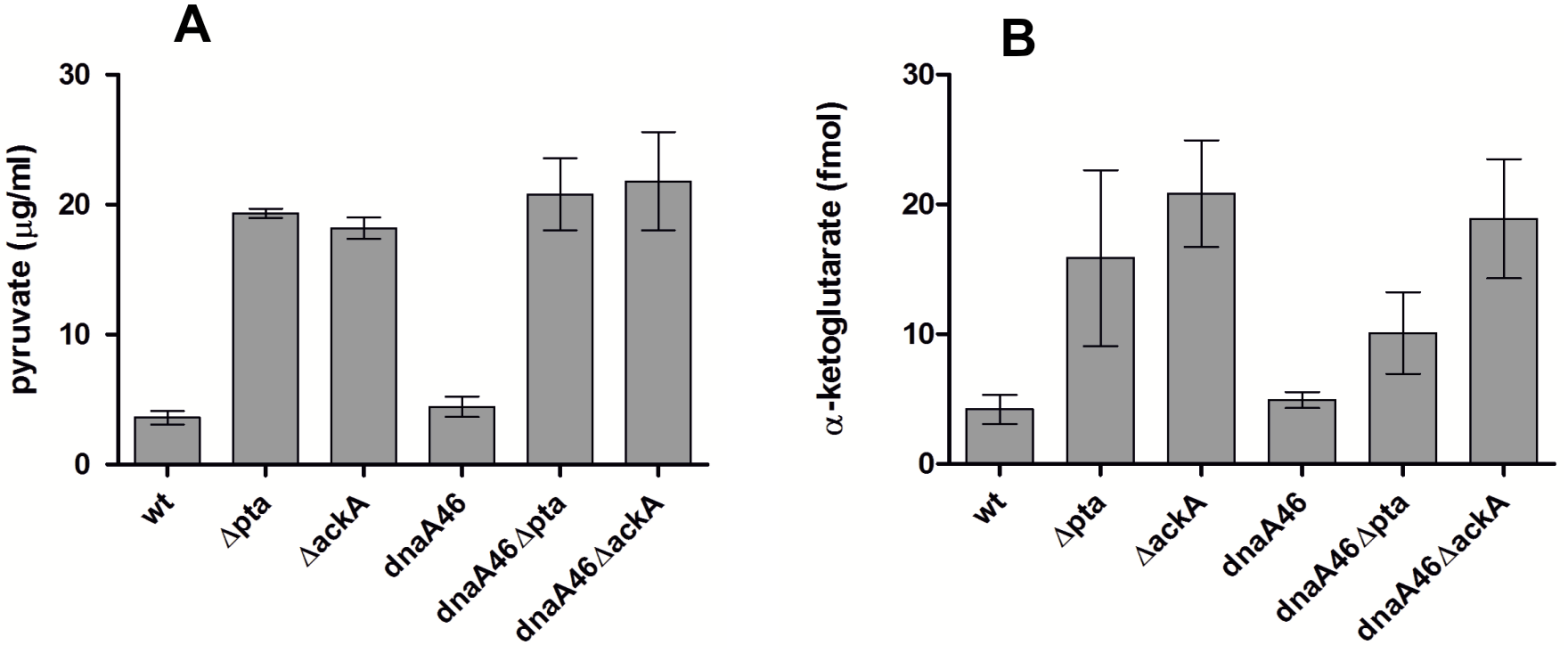
Intracellular accumulation of metabolites in the acetate overflow pathway mutants. A) pyruvate, B) 2-oxoglutarate. Concentration of metabolites was measured as described in Materials and Methods. Data represents mean ± SEM of two independent experiments. The values present metabolites’ concentration in the cellular extracts prepared from an equal bacterial mass but not their concentration in the bacterial cell volume.

Next, we aimed to determine the changes in the key metabolites which could be correlated with transcriptomic profile of the *pta* and *ackA* mutants, and that underlie the suppression of the replication initiator defects. The lack of the acetate overflow pathway activity may result in the deficit of the free CoA-SH [49] or imbalance of the NADH/NAD ratio, which both could lead to accumulation of pyruvate and 2-oxoglutarate due to limitation of performance of KGDH and PDH, both of which require free CoA-SH as a cofactor and contain the LpdA subunit, sensitive to the redox ratio [53, 54]. Therefore, we assayed the intracellular levels of pyruvate and 2-oxoglutarate in the *pta* and *ackA* strains. The single metabolic mutants accumulated pyruvate and 2-oxoglutarate, similarly to their *dnaA46* derivatives (Fig 6A and 6B, respectively; Fig E in S1 File).

Taken together, our results indicate that, under nutritional conditions supporting suppression of the *dnaA46* phenotypes, strains lacking the *pta* and *ackA* genes upregulate many stress and starvation response genes, whereas transcription profile of the CCM genes resembles that of strains grown on a poor carbon source like acetate. Changes in the level of pyruvate and 2-oxoglutarate may account for the increased expression of the stress and starvation-response genes in the acetate overflow mutants, due to these metabolites’ role in the synthesis of amino acids or function of the PTS system, since both the pyruvate/PEP ratio and the 2-oxoglutarate level regulate substrate uptake by the PTS system.

### Stress response factors are involved in the suppression of DnaA46 defects in the Δ*pta* and Δ*ackA* strains

**Fig 7:**
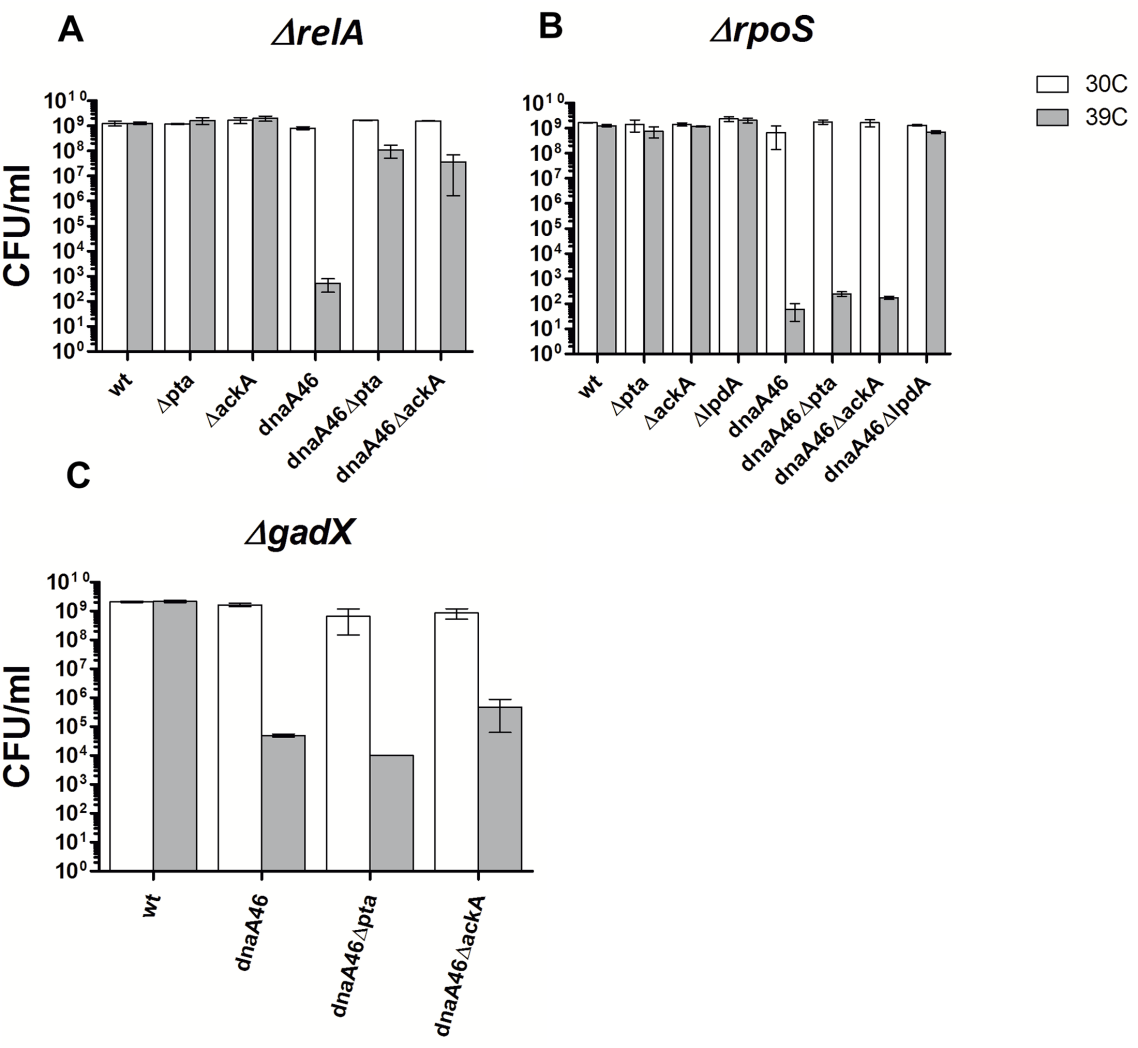
The influence of *rpoS* (A), *relA* (B) and *gadX* (C) on the suppression of the *dnaA46* temperature-sensitive growth by mutations in the acetate overflow pathway or *lpd* genes. Bacteria were grown at 30°C in LB medium to early exponential phase, subsequently serial dilutions were prepared and plated on LB plates at 30 and 39°C. Growth was estimated by CFU. Data represents mean ± SEM of three independent experiments.

Our results have demonstrated that the mutants with defective Pta-AckA pathway, display an increased level of transcripts of the *rpoS*-dependent genes when grown in LB medium. The increased expression of the genes regulated by σ^S^ and ppGpp in the strains lacking Pta or AckA prompted us to check whether suppression of the *dnaA46* temperature-sensitive growth depends on the *rpoS*, *relA* and *spoT* gene function. The required deletion mutants were constructed by P1 transduction and their ability to form colonies was monitored under permissive and restrictive conditions. Growth of the *dnaA46 Δpta ΔrelA* and *dnaA46 ΔackA ΔrelA* strains at the restrictive temperature was somewhat decreased in comparison to their *relA*+ counterparts under analogous conditions, however, it was still much higher than that of the *dnaA46 ΔrelA* (Fig 7A). Several attempts to create a quadruple mutant with deleted *spoT* were unsuccessful. Although this result requires further verification, it may suggest that *dnaA46 Δpta ΔrelA* and *dnaA46 ΔackA ΔrelA* cells are dependent on the *spoT* function or at least a minimal level of ppGpp even at the permissive temperature. Deletion of the *rpoS* gene, on the other hand, resulted in abolishment of suppression of the *dnaA46* temperature-sensitive phenotype by the dysfunction of the acetate overflow pathway (Fig 7B).

Since one of the most prominent features in the transcriptomic patterns of the strains with defective acetate overflow pathway is activation of many genes belonging to the GadX/GadW regulon which is responsible for survival under acidic conditions, (Fig 6, Table A in S1 File), we tested the effect of deletion of the *gadX* gene on suppression of the *dnaA46* temperature-sensitivity. Both, in the case of the *dnaA46 ΔptaΔ gadX* and *dnaA46 ΔackA ΔgadX* triple mutants, the number of colonies formed at the restrictive temperature was 3 to 4 orders of magnitude lower than in the case of their *gadX*+ counterparts, and was similar to that observed for the *dnaA46* single mutant (Fig 7C). This result suggests that GadX-regulated pathway or pathways are important for suppression to occur. Interestingly, we were not able to delete *gadX* in the *pta* or *ackA* mutants or remove any of the acetate overflow pathway components in the *gadX* background, despite several efforts. Further investigation is required to verify whether the combination of these mutations may be synthetically lethal or inhibitory for growth in the presence of the wild-type *dnaA* allele.

To summarize, the results presented in this study show that factors involved in stress response, i.e. σ^S^, GadX, and most likely ppGpp as well, are involved in the intracellular changes leading to suppression of the *dnaA46* phenotypes by the lack of the Pta or AckA function. This result is in agreement with our previous finding that the suppression occurs during fast growth under acetogenic conditions, but not during growth on glycerol as a sole carbon source, since perturbed growth and increased expression of some of the *rpoS*-dependent genes was previously reported to occur in LB but not in the glycerol minimal medium [43].

### Changes in the pyruvate dehydrogenase activity and acetate metabolism influence activity of the defective DnaA46 protein

Taking into account the observed accumulation of pyruvate and 2-oxoglutarate by the *pta* and *ackA* mutants, and the role of the acetate overflow pathway in maintaining homeostasis of Acetyl-CoA/CoA-SH and NADH/NAD ratio, we speculated that the increased levels of the metabolites may be evoked by a limited performance of the PDH or KGDH enzyme complex. Therefore, we tested whether the lack of the E3 subunit, encoded by *lpd*, could suppress the temperature-sensitivity of the *dnaA46* strain. Indeed, the *dnaA46 lpd* double mutant formed colonies at the restrictive temperature and their growth under these conditions was faster than in the case of inactivation of the acetate pathway (Table 1). Suppression was also observed for the *aceE* mutant, devoid of the E1 subunit of PDH (Table 1).

**Table 1:** Effect of deletion of the genes encoding pyruvate dehydrogenase subunits on growth of the *dnaA46* strain at the restrictive temperature.

Strain	Plating efficiency at 39°C
*dnaA46*	0.00015±0.0002
*dnaA46 lpdA*	1.8±0.2
*dnaA46 aceE*	0.8±0.79

Results are presented as a ratio of CFU obtained for the strains at restrictive and permissive temperature. Data represents mean ± SEM of three independent experiments.

Since accumulation of pyruvate is a common feature of all of the tested strains containing metabolic defects leading to suppression of the *dnaA46* temperature-sensitivity (*pta*, *ackA*, *lpd*, *aceE*), we tested whether supplementation of the culture medium with pyruvate allows for growth of the *dnaA46* mutant at the restrictive temperature. However, in a buffered medium, sodium pyruvate (even at a concentration as high as 100 mM) caused only minor improvement of growth of the *dnaA46* strain at the elevated temperature (Table 2).

**Table 2:** Effect of pyruvate and acetate on growth of the *dnaA46* strain at the restrictive temperature.

Concentration of metabolite	Plating efficiency at 39°C
-	0.00015±0.0002
50 mM sodium pyruvate	0.00015±0.0009
100 mM sodium pyruvate	0.0012±0.003
20 mM potassium acetate	1.28±0.3
50 mM potassium acetate	1.28±1

Results are presented as a ratio of CFU obtained for the strains at restrictive and permissive temperatures. Bacteria were grown in a buffered LB agar supplemented with indicated amounts of pyruvate and acetate. Data represents mean ± SEM of three independent experiments.

This result suggests that the reduced PDH complex activity, or the flow through alternative pathways, activated as a compensation of the lack of the PDH function, but not higher level of pyruvate *per se*, may be the cause of the decreased temperature sensitivity of the *dnaA46* strain growth in the presence of *pta* or *ackA* as well as the double *pta ackA* mutations.

**Fig 8:**
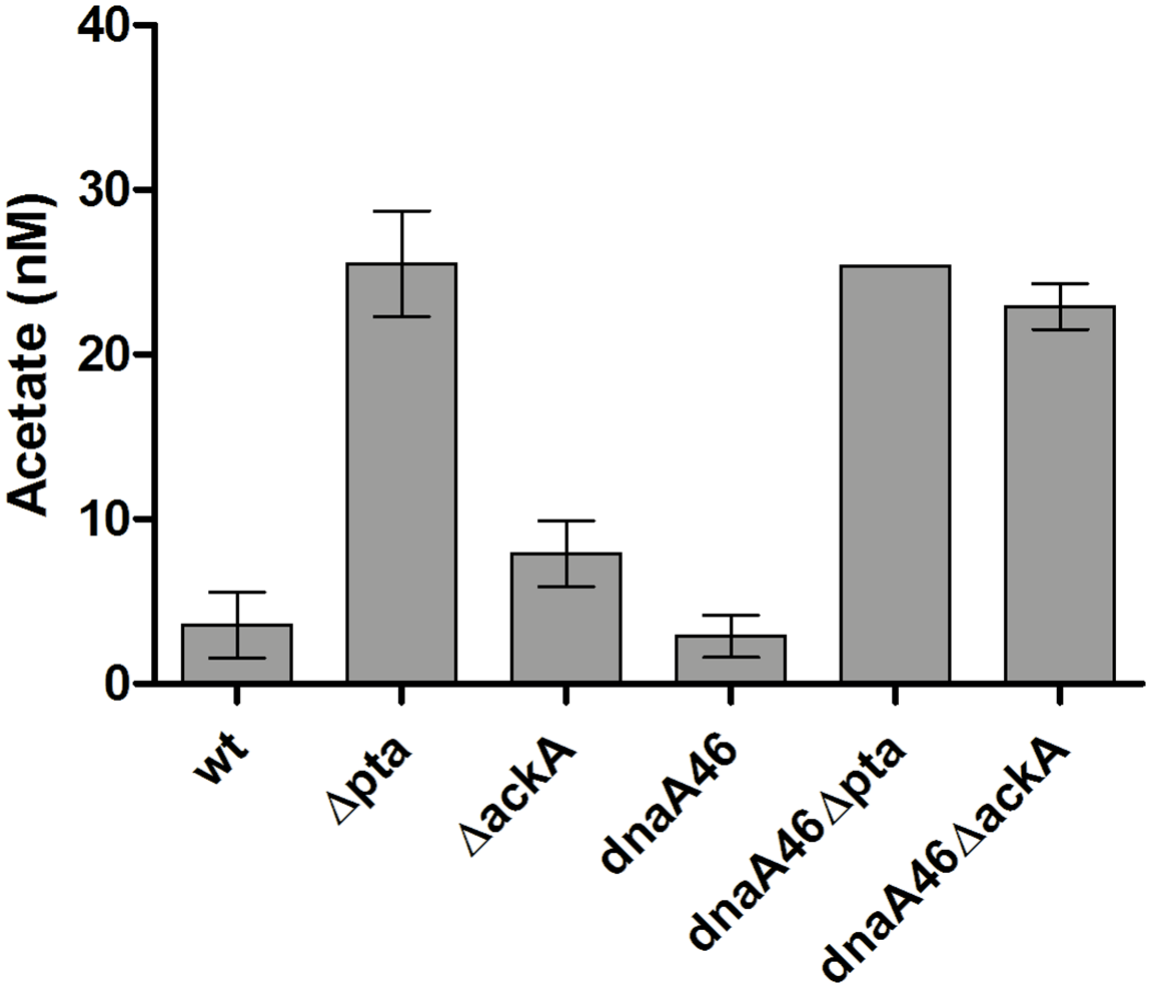
Intracellular accumulation of acetate in the *pta* and *ackA* mutants. Concentration of metabolites was measured as described in Materials and Methods. Data represents mean ± SEM of two independent experiments.

Transcription profile observed for both the *dnaA46 Δpta* and *dnaA46 ΔackA* strains resembled that of *E*. *coli* cells grown on acetate as a sole carbon source (Fig 4) [50]. Moreover, cells devoid of the activity of the lipoamide dehydrogenase also showed upregulation of the alternative pathway for acetyl-CoA generation, used during assimilation of acetate, namely the acetate synthetase (Acs) [55], suggesting that the flux through the CCM pathways may be similar in the case of the tested suppressor mutants (*pta*, *ackA*, *lpd*, *aceE*) and acetate-grown cells. Therefore, we tested whether the presence of acetate in the culture medium could affect colony formation of the *dnaA46* mutant at the restrictive temperature. Indeed, addition of moderate to high amounts of acetate allowed the replication initiation mutant to grow at the increased temperature (Table 2). This result suggests that suppression of the *dnaA46* defects may be related to this specific metabolic profile. Interestingly, high intracellular level of acetate was confirmed for both the single and double acetate overflow pathway mutants (Fig 8).

Still, the growth of the *dnaA46 Δlpd* mutant at the restrictive temperature was not dependent on the *rpoS* function (Fig 7B). Similarly, suppression of the temperature-sensitive phenotype by acetate was effective for the *dnaA46ΔrpoS* at 39° suggesting that the σ^S^ activity may be required for condition-dependent changes in the metabolic pathways, like these evoked by mutation in the Pta-AckA pathway.

### The mechanism of suppression is not linked to increased chromosomal DNA supercoiling or DnaA acetylation

In the search for the causes of suppression of the *dnaA46* phenotypes by the lack of acetate overflow pathway, we investigated a possible influence of alteration in chromosome structure or overall acetylation level on the growth of the *dnaA46* strain at the restrictive temperature.

**Fig 9:**
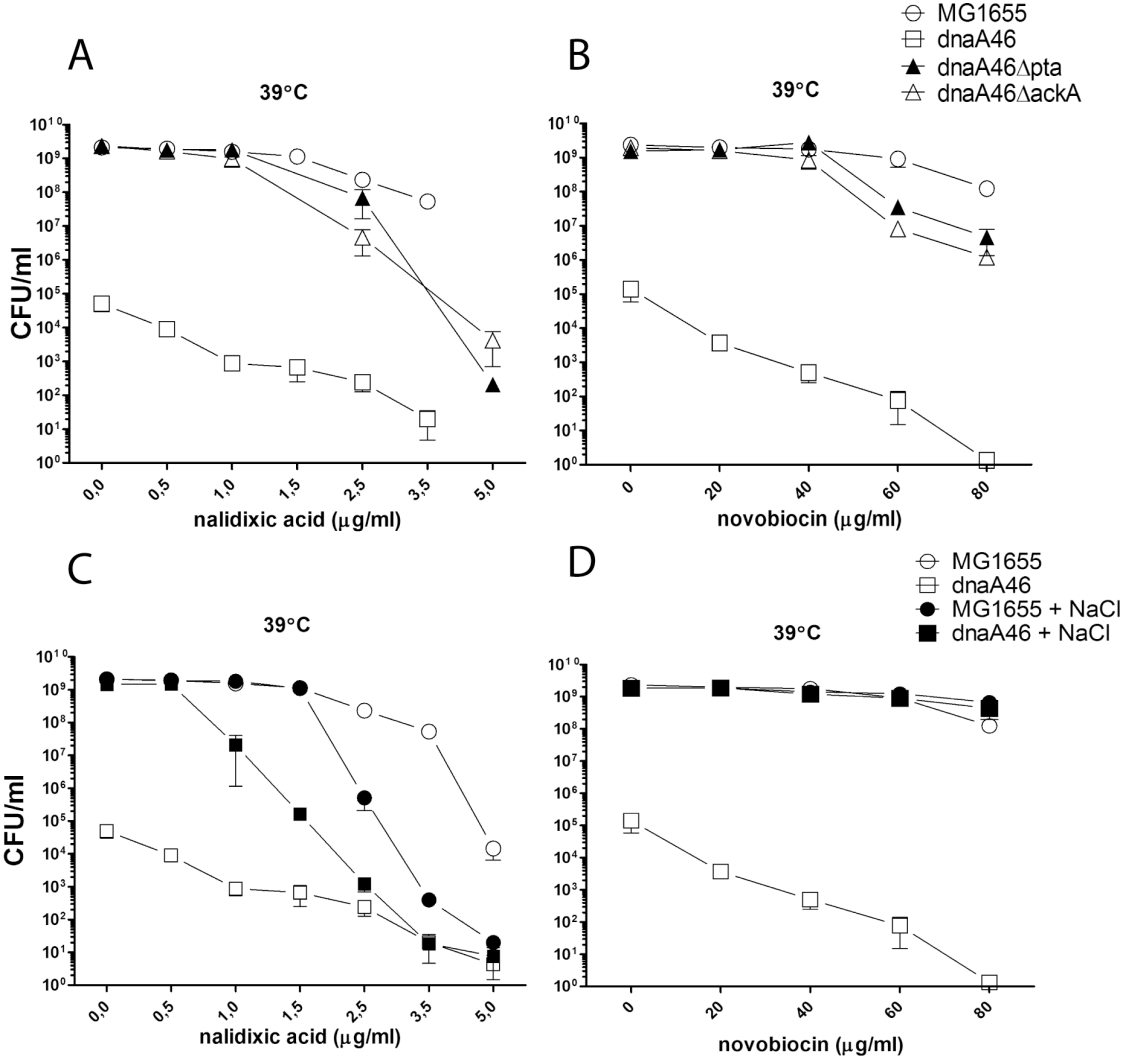
Sensitivity to gyrase inhibitors of the *dnaA46* strain lacking a part of acetate overflow pathway (AB) or growing in the presence of 0.5 M NaCl (CD). Indicated concentrations of nalidixic acid (AC) or novobiocin (BD) were used. Bacteria were grown on LB plates at 30 and 39°C and growth was estimated by CFU. Data represents mean ± SEM of three independent experiments.

In previous studies, suppressors of DnaA46 thermolability have been mapped to the *topA*, *rpoB* and *seqA* genes [56–58]. All of these genes can be linked to chromosomal DNA superhelicity or topology of DNA in the *oriC* region. The lack of *topA* or *seqA* increases the overall negative supercoiling of chromosomal DNA which could assist a defective DnaA protein in DNA unwinding and allow for initiation of DNA replication. In line with this proposal, as a stimulating effect of high negative superhelicity on the DnaA46 activity was shown *in vitro* [59]. Such mechanism of suppression was also proposed in the case of decreased temperature sensitivity of the *dnaA46* mutant grown in a medium containing 0.51M salt, which also results in an increased negative superhelicity of the chromosomal DNA [59]. Addition of nalidixic acid, an inhibitor of the DNA gyrase which introduces negative supercoils at the expense of ATP, was shown to reduce growth of the *dnaA46* strain at the restrictive temperature [59]. Due to involvement of the *pta* and *ackA* genes in cellular energetics, we hypothesized that the suppression of DnaA46 thermolability by deletion of these genes could result from an increase in the negative DNA supercoiling. Therefore, we tested the ability of the *dnaA46Δpta* and *dnaA46ΔackA* strains to form colonies at the restrictive temperature in the presence of increasing concentrations of nalidixic acid and another gyrase inhibitor, novobiocin, which competes with ATP-binding by the gyrase. Nalidixic acid, as in the case of suppression by high salt concentration, reduced the number of colonies formed by the *dnaA46* strain at the restrictive temperature (Fig 9A and 9C). Novobiocin in turn had little effect on the efficiency of suppression of the *dnaA46* temperature-sensitive growth by metabolic mutations and had an even lesser effect on suppression caused by a high salt concentration (Fig 9B and 9D), although coumermycin antibiotics were previously shown to effectively cause DNA relaxation *in vivo* during osmotic shock. Thus, we conclude that the decreased thermolability of initiation of the DNA replication in the *dnaA46* strain, both in the presence of metabolic mutations and high salt concentration, is not dependent on the gyrase activity. In agreement with these results, in the transcriptomic patterns of both *dnaA46 Δpta* and *dnaA46 ΔackA* double mutants, we noted an enhanced activity of promoters preferring DNA relaxation (data not shown). Promoters preferring high or low level of negative superhelicity have been identified in previous studies [60]. These results also corroborate induction of genes belonging to the regulon controlled by σ^S^, which was shown to depend on the DNA relaxation [61]. In the case of suppression of the *dnaA46* temperature-sensitive growth by high medium osmolarity, the lack of requirement for the *rpoS* function (data not shown) and insensitivity to inhibition by coumermycin antibiotics may also suggest that metabolic alterations evoked by high salt are responsible for the suppression.

Alterations in the AcCoA metabolism could lead to changes in the level and profile of intracellular protein modification by lysine acetylation. The overall protein acetylation status differs dramatically between the *ptaA* and *ackA* strains, due to accumulation of an acetyl group donor–acetyl phosphate in the latter strain and the lack of it in the former [39, 40]. We reasoned, however, that acetylation by YfiQ, the main acetyltransferase identified so far in *E*. *coli* [62], could be decreased in all *dnaA46* suppressor strains, leading to reduction of an inhibitory acetylation of a positive replication regulator. Indeed, deletion of the *yfiQ* gene led to an efficient suppression of the *dnaA46* temperature-sensitive growth (Table 3). Therefore, we increased acetylation level in the *dnaA46 Δpta* and *dnaA46 ΔackA* strains by deleting *cobB*, a gene encoding the NAD-dependent protein lysine-deacetylase which removes acetyl groups added via the *yfiQ* and acetyl phosphate-dependent pathways. However, the absence of *cobB* had no influence on the growth of the *dnaA46 Δpta* and *dnaA46 ΔackA* strains at an elevated temperature (Table 3). This result suggests that the suppression of the DnaA46 defect is most likely not dependent on a decrease of the *yfiQ*-dependent acetylation in the *pta* and *ackA* strains, whereas suppression by the lack of *yfiQ* operates via an independent mechanism. Specifically, it could be related to reversible acetylation of DnaA Lys178, necessary for ATP binding by DnaA [34]. Even though the DnaA46 defect relies on the low affinity for ATP and ADP, and thus it could be insensitive to the Lys178 acetylation status, it has been shown that in the presence of chaperone proteins and a high ATP level, DnaA46-ATP complex formation may take place [35]. In addition, *dnaA46* temperature-sensitivity can be suppressed by overexpression of the defective DnaA protein [63]. It is therefore possible that a fraction of DnaA46 is associated with ATP *in vivo*, and this fraction could be increased in the absence of YfiQ-dependent acetylation, leading to suppression. Alternatively, acetylation could additionally negatively influence the DnaA46 conformation, irrespective of the effect on ATP binding, and its removal could enhance the fraction of the active protein.

**Table 3:** Effect of deletion of the *yfiQ* and *cobB* genes on the growth of the *dnaA46* strain and its derivatives with disrupted acetate overflow pathway at the restrictive temperature.

Strain	Plating efficiency at 39°C
*dnaA46*	2.86 x 10−^6^ ± 0.12
*dnaA46 ΔyfiQ*	0.14 ± 0.22
*dnaA46 ΔyfiQ Δpta*	0.44 ± 0.16
*dnaA46 ΔyfiQ ΔackA*	0.56 ± 0.32
*dnaA46 ΔcobB*	1.52 x 10−^5^ ± 0.22
*dnaA46 ΔcobB Δpta*	0.39 ± 0.19
*dnaA46 ΔcobB ΔackA*	0.14 ± 0.17

Results are presented as a ratio of CFU obtained for the strains at restrictive and permissive temperatures. Data represents mean ± SEM of three independent experiments.

## Discussion

In this work we have shown that the suppression of *dnaA46* phenotypes by the absence of a part, or the whole acetate overflow pathway, depends on the growth conditions and occurs during fast growth in an acetogenic medium but not when glycerol is used as a sole carbon source (Fig 1). Furthermore, our results reveal that in a rich medium, strains devoid of *pta* or *ackA* upregulate a significant fraction of the *rpoS*-dependent genes (Fig 5, Table A in S1 File), corroborating results presented earlier by others [43] which had indicated an elevated level of some stress response-related proteins in the *pta-ackA* strain when grown in LB but not in a minimal medium with glycerol [43]. Among the upregulated genes, we also identified a high representation of genes jointly regulated by σ^S^ and ppGpp (Table A in S1 File). Overall transcription profile of the CCM genes in the *pta* and *ackA* mutants was similar to that of the acetate-grown cells, mainly due to upregulation of genes encoding: the acetate synthetase (Acs), the TCA cycle enzymes, some of the anaplerotic pathways’ enzymes utilized under gluconeogenetic conditions, as well as some transporters of alternative carbon sources (Fig 4, Table A in S1 File) [50]. However no significant changes in the RNA level of *dnaA* or its regulators, which could easily explain the suppressor phenotype, were detected. Therefore, we propose that the activity of metabolic enzymes or the level of metabolites, specific for the *pta* and *ackA* strain grown in LB is responsible for the observed suppression. In line with this hypothesis, we have found that addition of acetate also suppresses the *dnaA46* temperature-sensitive growth (Tables 1 and 2), similarly to the lack of Lpd or other subunits of PDH, which results in changes in the metabolism that in many aspects resembles that of the *pta* and *ackA* mutants. Namely, cells devoid of the Lpd or AceE activity also show upregulation of the alternative pathway for acetyl-CoA generation, utilized during assimilation of acetate, like acetate synthetase (Acs) or glyoxylate shunt [50], as well as an increased level of σ^S^ and ppGpp [64]. Moreover, we have shown that the *rpoS* and *gadX* activities are required in the *dnaA46pta* and *ackA* strains for alleviation of the temperature-sensitivity. Induction of the stress response could account for the suppression of the *dnaA46* thermal sensitivity, since the low affinity of the DnaA46 protein for ATP/ADP does not result from the change in amino acid residues that are directly involved in the nucleotide binding, but rather results from an effect on protein conformation, which can be rescued by the DnaK (heat-shock induced, σ^32^-dependent) chaperone protein *in vitro* [35]. Thus, production of the stress-induced chaperone proteins could account for maintaining of thereplication activity by the defective protein. However, *rpoS* was required for the suppression of *dnaA46* thermolability in the case of *pta* or *ackA* mutants, but not *lpd* and *aceE*. We suggest that in the *pta* and *ackA* mutants, σ^S^ and *gadX* are necessary for the specific suppressor metabolic profile to occur rather than suppression to result from any single *rpoS*-dependent gene product function. For instance, it has been shown that deletion of *rpoS* strongly induces expression of the PDH genes in *E*. *coli* during exponential phase of growth and GadX also regulates some genes associated with the nitrogen metabolism [65, 66] Alternatively, the conditions for suppression may be weaker in the *pta* and *ackA* strains and require additional, *rpoS*-dependent functions.

In this work, we have also excluded the global increase in negative supercoiling as a potential mechanism of suppression of the DnaA46 defects by the lack of *pta* and *ackA* activity; our results also argue against changes in the DnaA acetylation as the underlying cause. The results call also into question the mechanism of suppression of the *dnaA46* temperature-sensitivity by high salt concentration which was proposed to operate via increased negative DNA supercoiling (Fig 9). High medium osmolarity results in an elevated level of the DnaK protein [67], and also leads to significant changes in the cellular metabolism [68] which could alleviate the loss of activity by DnaA46.

The reasons for the observed shift in the global gene expression pattern in the absence of the acetate overflow pathway activity are not completely understood. Accumulation of pyruvate implies that the flux to pyruvate exceeds the capacity of the PDH complex to synthesize acetyl-CoA. It was previously suggested that the PDH activity is partially restrained in the *pta* and *pta-ackA* strains due to the accumulation of acetyl-CoA and the lack of free CoA-SH, since expression of a heterologous acetyl-CoA consuming pathway had abolished excretion of the unusual metabolites (pyruvate, lactate, glutamate) by the *pta* strain, restoring the wild-type exometabolome profile [49]. However, the accumulation of acetyl-CoA in strains lacking Pta-AckA pathway was not confirmed [41]. Another possibility is that the redox imbalance in these strains could also pertain to pyruvate accumulation, due to the increased NADH/NAD ratio which is inhibitory for the PDH activity (Fig D2 in S1 File) [53]. The same reason could also account for pyruvate accumulation in the *ackA* strain or, alternatively, acetylation of LpdA or another PDH subunit could alter this complex’s function. High levels of acetyl phosphate, present in *ackA* mutants, result in global enhancement of lysine-acetylation, whereas LpdA as well as AceE are among the most highly acetylated proteins under these conditions [69]. Pyruvate and 2-oxoglutarate are important for the function of the PTS system, constitute precursors of the synthesis of amino acids and 2-oxoglutarate also plays a role in coordination of the nitrogen and carbon status, and thus their imbalance may lead to induction of starvation and stress responses [70]. Downregulation of several genes involved in the synthesis and transport of branched amino-acids (*ilvB*, *ilvG*–nonfunctional in *E*. *coli* K-12, *livKH*), derived from pyruvate is consistent with this scenario (Fig D in S1 File).

To summarize, our results suggest that changes in the flux of the pyruvate-acetyl CoA-acetate node influence the initiation of DNA replication. Elucidation of the exact mechanism of this link between metabolism and replication requires further investigation, however several possible scenarios can be proposed. The most probable is the one that changes in the level of metabolite or activity of metabolic enzyme, facilitate the functional complex formation by DnaA, directly or via an effect exerted on another regulatory protein. Changes in the level of metabolites may lead to an altered post-translational modification of proteins controlling the replication initiation step or these proteins may themselves interact with metabolites affecting their activity, like for instance, DiaA protein which contains a sugar isomerase domain of unknown function [71]. Secondly, metabolic enzymes may directly affect the activity of replication proteins, similarly to their role in the control of a cell division process. Our results suggests that alterations in the PDH complex activity may account for restoration of DNA replication in the *dnaA46* mutant, and PDH could be a candidate protein directly involved in the control of other processes. However, we excluded the increased pyruvate level as underlying the suppressory effect of the tested mutations, since supplementation of the culture medium even with high concentration of pyruvate has a minor effect on the *dnaA46* strain growth at the restrictive temperature (Table 2). Interestingly, in *B*. *subtilis*, the dihydrolipoamide acetyltransferase (E2) subunit of PDH was identified as a membrane-associated factor inhibiting DNA replication, probably via binding to the AT-rich region present at the origin of replication [72, 73]. Moreover, in the same organism, E2 was identified as an interacting partner of the DnaG primase in a study employing yeast two-hybrid system [74]. In addition, most recent studies suggest involvement of the E1 subunit of PDH in the cell division of *B*. *subtilis* by its effect on the Z-ring formation [20]. These results strongly suggest coupling of the pyruvate metabolism to the DNA replication and cell cycle in this bacterium. Such a relation has not been uncovered so far for *E*. *coli*, but our results support a possibility of a similar mechanisms operating in this case as well. Interestingly, in a high-throughput protein-protein interaction study, LpdA was found in a complex with the DnaA protein, and AceE was found to interact with DnaB [75].

Another possible mechanism of suppression could rely on the altered membrane properties in the metabolic mutants. The DnaA protein binds to the membrane, and the membrane phospholipids stimulate rejuvenation of DnaA-ATP [18, 76]. Moreover, expression of genes related to the membrane synthesis was affected in the *dnaA46* mutant at the restrictive temperature [77]. In our studies, both, the *dnaA46pta* and *dna46ackA* mutant, showed an increased sensitivity to a variety of chemicals and antibiotics as well as an enhanced leakage of Mg^2+^ from the cells, in comparison to the single *dnaA46*, *pta* and *ackA* mutants, even at the permissive temperature (data not shown) which suggests broad changes in the membrane function. The apparent incompatibility of these mutants with *spoT* deletion could also suggest changes in the lipid synthesis, since mutations in *aceE* and *fabH*, which affect synthesis of membrane lipids, were found to be synthetically lethal with a *spoT* deletion [64, 78].

## Supporting information

S1 File (PDF)

## References

[1] Cooper S, Helmstetter CE. Chromosome replication and the division cycle of *Escherichia coli* B/r. J Mol Biol. 1968; 31: 519–40 4866337 10.1016/0022-2836(68)90425-7

[2] Donachie WD. Relationship between cell size and time of initiation of DNA replication. Nature. 1968; 219: 1077–9 4876941 10.1038/2191077a0

[3] Wallden M, Fange D, Lundius EG, Baltekin Ö, Elf J. The synchronization of replication and division cycles in individual *E*. *coli* cells. Cell. 2016; 166: 729–39 10.1016/j.cell.2016.06.052 27471967

[4] Leonard AC, Grimwade JE. The orisome: structure and function. Front Microbiol. 2015; 6: 545 10.3389/fmicb.2015.00545 26082765 PMC4451416

[5] Zheng H, Ho PY, Jiang M, Tang B, Liu W, Li D, et al Interrogating the Escherichia coli cell cycle by cell dimension perturbations. Proc Natl Acad Sci U S A. 2016; 113:15000–15005. 10.1073/pnas.1617932114 27956612 PMC5206551

[6] Likhoshvai VA, Khlebodarova TM. Mathematical modeling of bacterial cell cycle: the problem of coordinating genome replication with cell growth. J Bioinform Comput Biol. 2014; 12:1450009 10.1142/S0219720014500097 24969747

[7] Leonard AC, Grimwade JE. The orisome: structure and function. Front Microbiol. 2015; 6: 545 10.3389/fmicb.2015.00545 26082765 PMC4451416

[8] Katayama T, Kubota T, Kurokawa K, Crooke E, Sekimizu K. The initiator function of DnaA protein is negatively regulated by the sliding clamp of the *E*. *coli* chromosomal replicase. Cell. 1998; 94: 61–71 9674428 10.1016/s0092-8674(00)81222-2

[9] Kato J, Katayama T. Hda, a novel DnaA-related protein, regulates the replication cycle in *Escherichia coli*. EMBO J. 2001; 20: 4253–62 10.1093/emboj/20.15.4253 11483528 PMC149159

[10] Kasho K, Katayama T. DnaA binding locus datA promotes DnaA-ATP hydrolysis to enable cell cycle-coordinated replication initiation. Proc Natl Acad Sci U S A. 2013; 110: 936–41 10.1073/pnas.1212070110 23277577 PMC3549119

[11] Nozaki S, Yamada Y, Ogawa T. Initiator titration complex formed at *datA* with the aid of IHF regulates replication timing in *Escherichia coli*. Genes Cells. 2009; 14: 329–41 10.1111/j.1365-2443.2008.01269.x 19170757

[12] Saxena R, Fingland N, Patil D, Sharma AK, Crooke E. Crosstalk between DnaA protein, the initiator of *Escherichia coli* chromosomal replication, and acidic phospholipids present in bacterial membranes. Int J Mol Sci. 2013; 14: 8517–37 10.3390/ijms14048517 23595001 PMC3645759

[13] Fujimitsu K, Senriuchi T, Katayama T. Specific genomic sequences of *E*. *coli* promote replicational initiation by directly reactivating ADP-DnaA. Genes Dev. 2009; 23: 1221–33 10.1101/gad.1775809 19401329 PMC2685538

[14] Jameson KH, Wilkinson AJ. Control of Initiation of DNA Replication in Bacillus subtilis and *Escherichia coli*. Genes (Basel). 2017; 8: E22 28075389 10.3390/genes8010022 PMC5295017

[15] Speck C, Weigel C, Messer W. ATP- and ADP-DnaA protein, a molecular switch in gene regulation. EMBO J. 1999; 18: 6169–76 10.1093/emboj/18.21.6169 10545126 PMC1171680

[16] Saggioro C, Olliver A, Sclavi B. Temperature-dependence of the DnaA-DNA interaction and its effect on the autoregulation of dnaA expression. Biochem J. 2013; 449: 333–41 10.1042/BJ20120876 23092251

[17] Flåtten I, Fossum-Raunehaug S, Taipale R, Martinsen S, Skarstad K. The DnaA protein is not the limiting factor for initiation of replication in *Escherichia coli*. PLoS Genet. 2015; 11: e1005276 10.1371/journal.pgen.1005276 26047361 PMC4457925

[18] Chien AC, Zareh SK, Wang YM, Levin PA. Changes in the oligomerization potential of the division inhibitor UgtP co-ordinate *Bacillus subtilis* cell size with nutrient availability. Mol Microbiol. 2012; 86: 594–610. 10.1111/mmi.12007 22931116 PMC3480987

[19] Hill NS, Buske PJ, Shi Y, Levin PA. A moonlighting enzyme links *Escherichia coli* cell size with central metabolism. PLoS Genet. 2013; 9: e1003663 10.1371/journal.pgen.1003663 23935518 PMC3723540

[20] Monahan LG, Hajduk IV, Blaber SP, Charles IG, Harry EJ. Coordinating bacterial cell division with nutrient availability: a role for glycolysis. MBio. 2014; 5: e00935–14 10.1128/mBio.00935-14 24825009 PMC4030479

[21] Weart RB, Lee AH, Chien AC, Haeusser DP, Hill NS, Levin PA. A metabolic sensor governing cell size in bacteria. Cell. 2007; 130: 335–47. 10.1016/j.cell.2007.05.043 17662947 PMC1971218

[22] Beaufay F, Coppine J, Mayard A, Laloux G, De Bolle X, Hallez R. A NAD-dependent glutamate dehydrogenase coordinates metabolism with cell division in *Caulobacter crescentus*. EMBO J. 2015; 34: 1786–800. 10.15252/embj.201490730 25953831 PMC4516431

[23] Radhakrishnan SK, Pritchard S, Viollier PH. Coupling prokaryotic cell fate and division control with a bifunctional and oscillating oxidoreductase homolog. Dev Cell. 2010; 18: 90–101. 10.1016/j.devcel.2009.10.024 20152180

[24] Liu F, Qimuge, Hao J, Yan H, Bach T, Fan L, et al AspC-mediated aspartate metabolism coordinates the *Escherichia coli* cell cycle. PLoS One. 2014; 9: e922292014 10.1371/journal.pone.0092229 PMC3966765 24670900

[25] Shi Z, Xuan C, Han H, Cheng X, Wang J, Feng Y, Srinivas S, Lu G, Gao GF. Gluconate 5-dehydrogenase (Ga5DH) participates in *Streptococcus suis* cell division. Protein Cell. 2014; 5: 761–9. 10.1007/s13238-014-0074-8 24916441 PMC4180457

[26] Takada H, Fukushima-Tanaka S, Morita M, Kasahara Y, Watanabe S, Chibazakura T, Hara H, Matsumoto K, Yoshikawa H. An essential enzyme for phospholipid synthesis associates with the *Bacillus subtilis* divisome. Mol Microbiol. 2014; 91: 242–55. 10.1111/mmi.12457 24224907

[27] Haeusser DP, Levin PA. The great divide: coordinating cell cycle events during bacterial growth and division. Curr Opin Microbiol. 2008; 11: 94–9. 10.1016/j.mib.2008.02.008 18396093 PMC2397022

[28] Wang JD, Levin PA. Metabolism, cell growth and the bacterial cell cycle. Nat Rev Microbiol. 2009; 7: 822–7. 10.1038/nrmicro2202 19806155 PMC2887316

[29] Monahan LG, Harry EJ. You are what you eat: Metabolic control of bacterial division. Trends Microbiol. 2016; 24: 181–9. 10.1016/j.tim.2015.11.007 26690613

[30] Murray H, Koh A. Multiple regulatory systems coordinate DNA replication with cell growth in *Bacillus subtilis*. PLoS Genet. 2014; 10: e1004731 10.1371/journal.pgen.1004731 25340815 PMC4207641

[31] Jannière L, Canceill D, Suski C, Kanga S, Dalmais B, Lestini R, et al Genetic evidence for a link between glycolysis and DNA replication. PLoS One. 2007; 2: e447 10.1371/journal.pone.0000447 17505547 PMC1866360

[32] Maciąg M, Nowicki D, Janniere L, Szalewska-Pałasz A, Węgrzyn G. Genetic response to metabolic fluctuations: correlation between central carbon metabolism and DNA replication in *Escherichia coli*. Microb Cell Fact. 2011; 10: 19 10.1186/1475-2859-10-19 21453533 PMC3080795

[33] Maciąg M, Nowicki D, Szalewska-Pałasz A, Węgrzyn G. Central carbon metabolism influences fidelity of DNA replication in *Escherichia coli*. Mutat Res. 2012; 731: 99–106 10.1016/j.mrfmmm.2011.12.005 22198407

[34] Zhang Q, Zhou A, Li S, Ni J, Tao J, Lu J, et al Reversible lysine acetylation is involved in DNA replication initiation by regulating activities of initiator DnaA in *Escherichia coli*. Sci Rep. 2016; 6: 30837 10.1038/srep30837 27484197 PMC4971506

[35] Carr KM, Kaguni JM. The A184V missense mutation of the *dnaA5* and *dnaA46* alleles confers a defect in ATP binding and thermolability in initiation of *Escherichia coli* DNA replication. Mol Microbiol. 1996; 20: 1307–18 8809781 10.1111/j.1365-2958.1996.tb02649.x

[36] El-Mansi M. Free CoA-mediated regulation of intermediary and central metabolism: an hypothesis which accounts for the excretion of alpha-ketoglutarate during aerobic growth of *Escherichia coli* on acetate. Res Microbiol. 2005; 156: 874–9 10.1016/j.resmic.2005.04.008 16171983

[37] Vemuri GN, Altman E, Sangurdekar DP, Khodursky AB, Eiteman MA. Overflow metabolism in *Escherichia coli* during steady-state growth: transcriptional regulation and effect of the redox ratio. Appl Environ Microbiol. 2006; 72: 3653–61 10.1128/AEM.72.5.3653-3661.2006 16672514 PMC1472329

[38] Berríos-Rivera SJ, San KY, Bennett GN. The effect of NAPRTase overexpression on the total levels of NAD, the NADH/NAD+ ratio, and the distribution of metabolites in *Escherichia coli*. Metab Eng. 2002; 4: 238–47 12616693 10.1006/mben.2002.0229

[39] Schilling B, Christensen D, Davis R, Sahu AK, Hu LI, Walker-Peddakotla A, et al Protein acetylation dynamics in response to carbon overflow in *Escherichia coli*. Mol Microbiol. 2015; 98: 847–63 10.1111/mmi.13161 26264774 PMC4715485

[40] Weinert BT, Iesmantavicius V, Wagner SA, Schölz C, Gummesson B, Beli P, et al Acetyl-phosphate is a critical determinant of lysine acetylation in *E*. *coli*. Mol Cell. 2013; 51: 265–72 10.1016/j.molcel.2013.06.003 23830618

[41] Klein AH, Shulla A, Reimann SA, Keating DH, Wolfe AJ. The intracellular concentration of acetyl phosphate in *Escherichia coli* is sufficient for direct phosphorylation of two-component response regulators. J Bacteriol. 2007; 189: 5574–81 10.1128/JB.00564-07 17545286 PMC1951799

[42] Basan M, Hui S, Okano H, Zhang Z, Shen Y, Williamson JR, et al Overflow metabolism in *Escherichia coli* results from efficient proteome allocation. Nature. 2015; 528: 99–104 10.1038/nature15765 26632588 PMC4843128

[43] Kirkpatrick C, Maurer LM, Oyelakin NE, Yoncheva YN, Maurer R, Slonczewski JL. Acetate and formate stress: opposite responses in the proteome of *Escherichia co*li. J Bacteriol. 2001; 183: 6466–77 10.1128/JB.183.21.6466-6477.2001 11591692 PMC100143

[44] Baba T, Mori H. The construction of systematic in-frame, single-gene knockout mutant collection in *Escherichia coli* K-12. Methods Mol Biol. 2008; 416: 171–81. 10.1007/978-1-59745-321-9_11 18392967

[45] Gentry DR, Hernandez VJ, Nguyen LH, Jensen DB, Cashel M. Synthesis of the stationary-phase sigma factor sigma s is positively regulated by ppGpp. J Bacteriol. 1993;175: 7982–9. 8253685 10.1128/jb.175.24.7982-7989.1993 PMC206978

[46] Glinkowska M, Konopa G, Wegrzyn A, Herman-Antosiewicz A, Weigel C, Seitz H, et al The double mechanism of incompatibility between lambda plasmids and *Escherichia coli dnaA*(ts) host cells. Microbiology. 2001; 147: 1923–8. 10.1099/00221287-147-7-1923 11429468

[47] Jensen KF. The *Escherichia coli* K-12 "wild types" W3110 and MG1655 have an *rph* frameshift mutation that leads to pyrimidine starvation due to low *pyrE* expression levels. J Bacteriol. 1993; 175: 3401–3407 8501045 10.1128/jb.175.11.3401-3407.1993 PMC204738

[48] Rabinowitz JD, Kimball E. Acidic acetonitrile for cellular metabolome extraction from *Escherichia coli*. Anal Chem. 2007; 79: 6167–73 10.1021/ac070470c 17630720

[49] Chang DE, Shin S, Rhee JS, Pan JG. Acetate metabolism in a pta mutant of *Escherichia coli* W3110: importance of maintaining acetyl coenzyme A flux for growth and survival. J Bacteriol. 1999;181: 6656–63 10542166 10.1128/jb.181.21.6656-6663.1999 PMC94129

[50] Oh MK, Rohlin L, Kao KC, Liao JC. Global expression profiling of acetate-grown Escherichia coli. J Biol Chem. 2002; 277: 13175–83 10.1074/jbc.M110809200 11815613

[51] Wolfe AJ, Chang DE, Walker JD, Seitz-Partridge JE, Vidaurri MD, Lange CF, et al Evidence that acetyl phosphate functions as a global signal during biofilm development. Mol Microbiol. 2003; 48: 977–88. 12753190 10.1046/j.1365-2958.2003.03457.x

[52] Traxler MF, Zacharia VM, Marquardt S, Summers SM, Nguyen HT, Stark SE, et al Discretely calibrated regulatory loops controlled by ppGpp partition gene induction across the 'feast to famine' gradient in *Escherichia coli*. Mol Microbiol. 2011; 79: 830–45. 10.1111/j.1365-2958.2010.07498.x 21299642 PMC3073637

[53] Kim Y, Ingram LO, Shanmugam KT. Dihydrolipoamide dehydrogenase mutation alters the NADH sensitivity of pyruvate dehydrogenase complex of Escherichia coli K-12. J Bacteriol. 2008; 190: 3851–8 10.1128/JB.00104-08 18375566 PMC2395023

[54] Ojima Y, Suryadarma P, Tsuchida K, Taya M. Accumulation of pyruvate by changing the redox status in *Escherichia coli*. Biotechnol Lett. 2012; 34: 889–93 10.1007/s10529-011-0842-y 22215378

[55] Li M, Ho PY, Yao S, Shimizu K. Effect of lpdA gene knockout on the metabolism in Escherichia coli based on enzyme activities, intracellular metabolite concentrations and metabolic flux analysis by 13C-labeling experiments. J Biotechnol. 2006; 122: 254–66 10.1016/j.jbiotec.2005.09.016 16310273

[56] Louarn J, Bouché JP, Patte J, Louarn JM. Genetic inactivation of topoisomerase I suppresses a defect in initiation of chromosome replication in *Escherichia coli*. Mol Gen Genet. 1984; 195: 170–4. 6092846 10.1007/BF00332741

[57] Atlung T. Allele-specific suppression of *dnaA*(Ts) mutations by *rpoB* mutations in *Escherichia coli*. Mol Gen Genet. 1984; 197: 125–8. 6096668 10.1007/BF00327932

[58] Lu M., Campbell JL, Boye E, and Kleckner N. SeqA: a negative modulator of replication initiation in *E*. *coli*. Cell. 1994; 77: 413–426. 8011018 10.1016/0092-8674(94)90156-2

[59] Kondo T, Mima S, Fukuma N, Sekimizu K, Tsuchiya T, Mizushima T. Suppression of temperature-sensitivity of a *dnaA46* mutant by excessive DNA supercoiling. Biochem J. 2000; 348: 375–9. 10816432 PMC1221076

[60] Blot N, Mavathur R, Geertz M, Travers A, Muskhelishvili G. Homeostatic regulation of supercoiling sensitivity coordinates transcription of the bacterial genome. EMBO Rep. 2006; 7: 710–5 10.1038/sj.embor.7400729 16799466 PMC1500834

[61] Bordes P, Conter A, Morales V, Bouvier J, Kolb A, Gutierrez C. DNA supercoiling contributes to disconnect sigma S accumulation from sigma S-dependent transcription in *Escherichia coli*. Mol Microbiol. 2003; 48: 561–71. 12675812 10.1046/j.1365-2958.2003.03461.x

[62] Wolfe AJ. Bacterial protein acetylation: new discoveries unanswered questions. Curr Genet. 2016; 62: 335–41 10.1007/s00294-015-0552-4 26660885 PMC4826803

[63] Nyborg M, Atlung T, Skovgaard O, Hansen FG. Two types of cold sensitivity associated with the A184—>V change in the DnaA protein. Mol Microbiol. 2000; 35: 1202–10. 10712700 10.1046/j.1365-2958.2000.01790.x

[64] Battesti A, Majdalani N Gottesman S. Stress sigma factor RpoS degradation and translation are sensitive to the state of central metabolism. Proc Natl Acad Sci U S A. 112; 5159–64 (2015) 10.1073/pnas.1504639112 25847996 PMC4413282

[65] Rahman M, Hasan MR, Oba T, Shimizu K. Effect of rpoS gene knockout on the metabolism of *Escherichia coli* during exponential growth phase and early stationary phase based on gene expressions, enzyme activities and intracellular metabolite concentrations. Biotechnol Bioeng. 2006; 94: 585–95 10.1002/bit.20858 16511888

[66] Hommais F, Krin E, Coppée JY, Lacroix C, Yeramian E, Danchin A, et al GadE (YhiE): a novel activator involved in the response to acid environment in *Escherichia coli*. Microbiology. 2004;150: 61–72 10.1099/mic.0.26659-0 14702398

[67] Meury J, Kohiyama M. Role of heat shock protein DnaK in osmotic adaptation of *Escherichia coli*. J Bacteriol. 1991; 173: 4404–10. 2066337 10.1128/jb.173.14.4404-4410.1991 PMC208102

[68] Sévin DC, Sauer U. Ubiquinone accumulation improves osmotic-stress tolerancein *Escherichia coli*. Nat Chem Biol. 2014;10: 266–72 10.1038/nchembio.1437 24509820

[69] Kuhn ML, Zemaitaitis B, Hu LI, Sahu A, Sorensen D, Minasov G, et al Structural, kinetic and proteomic characterization of acetyl phosphate-dependent bacterial protein acetylation. PLoS One. 2014; 9: e94816 10.1371/journal.pone.0094816 24756028 PMC3995681

[70] Huergo LF, Dixon R. The emergence of 2-oxoglutarate as a master regulator metabolite. Microbiol Mol Biol Rev. 2015; 79: 419–35 10.1128/MMBR.00038-15 26424716 PMC4651028

[71] Keyamura K, Fujikawa N, Ishida T, Ozaki S, Su'etsugu M, Fujimitsu K, et al The interaction of DiaA and DnaA regulates the replication cycle in *E*. *coli* by directly promoting ATP DnaA-specific initiation complexes. Genes Dev. 2007; 21: 62083–99 10.1101/gad.1561207 PMC1948862 17699754

[72] Laffan JJ, Firshein W. Origin-specific DNA-binding membrane-associated protein may be involved in repression of initiation of DNA replication in *Bacillus subtilis*. Proc Natl Acad Sci U S A. 1988; 85: 7452–6. 3140241 10.1073/pnas.85.20.7452 PMC282209

[73] Stein A, Firshein W. Probable identification of a membrane-associated repressor of *Bacillus subtilis* DNA replication as the E2 subunit of the pyruvate dehydrogenase complex. J Bacteriol. 2000; 182: 2119–24 10735853 10.1128/jb.182.8.2119-2124.2000 PMC111259

[74] Noirot-Gros MF, Dervyn E, Wu LJ, Mervelet P, Errington J, Ehrlich SD, et al An expanded view of bacterial DNA replication. Proc Natl Acad Sci U S A. 2002; 99: 8342–7 10.1073/pnas.122040799 12060778 PMC123069

[75] Butland G, Peregrín-Alvarez JM, Li J, Yang W, Yang X, Canadien V, et al Interaction network containing conserved and essential protein complexes in *Escherichia coli*. Nature. 2005; 433: 531–7 10.1038/nature03239 15690043

[76] Aranovich A, Braier-Marcovitz S, Ansbacher E, Granek R, Parola AH, Fishov I. N-terminal-mediated oligomerization of DnaA drives the occupancy-dependent rejuvenation of the protein on the membrane. Biosci Rep. 2015; 35(5) 10.1042/BSR20150175 PMC4721551 26272946

[77] Ohba A, Mizushima T, Katayama T, Sekimizu K. Amounts of proteins altered by mutations in the *dnaA* gene of *Escherichia coli*. FEBS Lett. 1997; 404: 125–8 9119048 10.1016/s0014-5793(97)00108-7

[78] Yao Z, Davis RM, Kishony R, Kahne D, Ruiz N. Regulation of cell size in response to nutrient availability by fatty acid biosynthesis in *Escherichia coli*. Proc Natl Acad Sci U S A. 2012; 109: E2561–8 10.1073/pnas.1209742109 22908292 PMC3458391

