# Supplementary material for: Suppression of the Escherichia coli dnaA46 mutation by changes in the activities of the pyruvate-acetate node links DNA replication regulation to central carbon metabolism
Source: PLoS One. 2017 Apr 27;12(4):e0176050. doi: 10.1371/journal.pone.0176050 (PMC5407757; doi:10.1371/journal.pone.0176050)
Supplement: S1 File — (PDF) [file pone.0176050.s001.pdf]

Supporting information

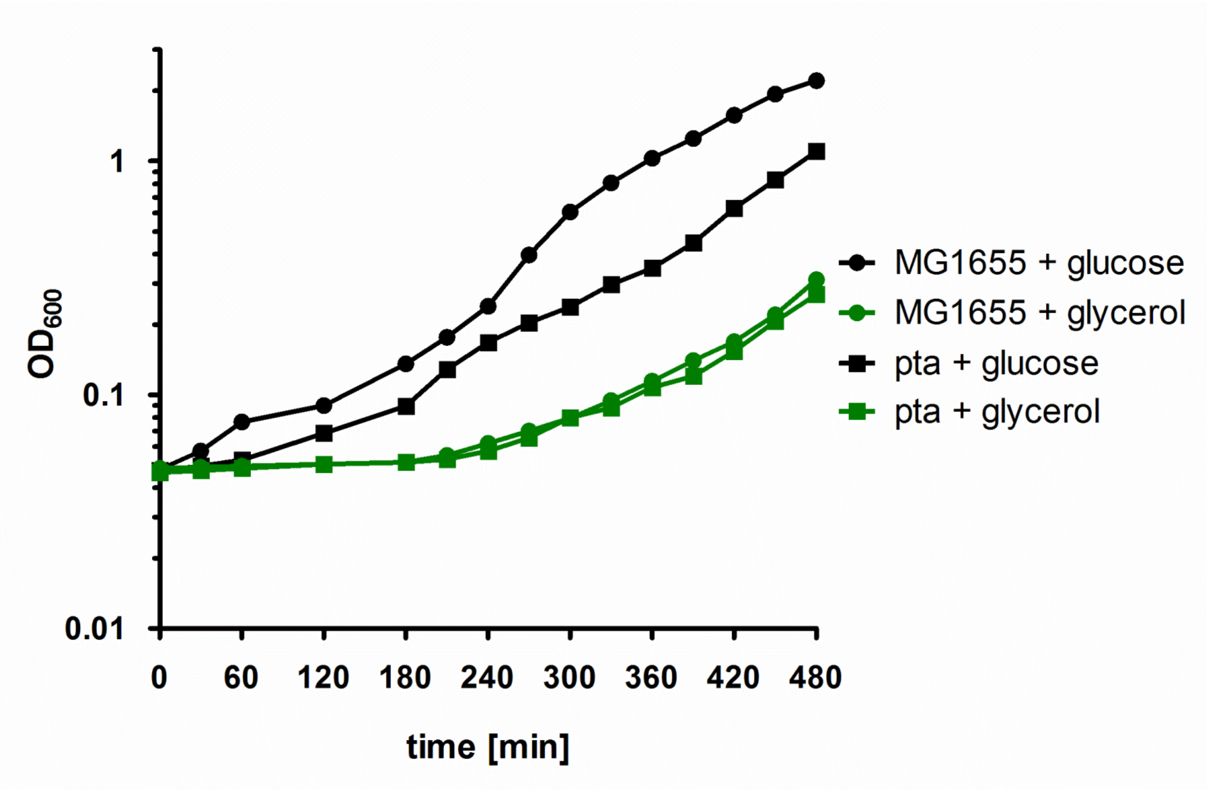

Fig A. The negative effect of *pta* deletion on *E. coli* growth is much more pronounced in the presence of glucose as a sole carbon source than in glycerol. Overnight cultures were prepared in the LB medium, diluted 1:1000 in a fresh minimal medium and cultivated with shaking at 30°C.

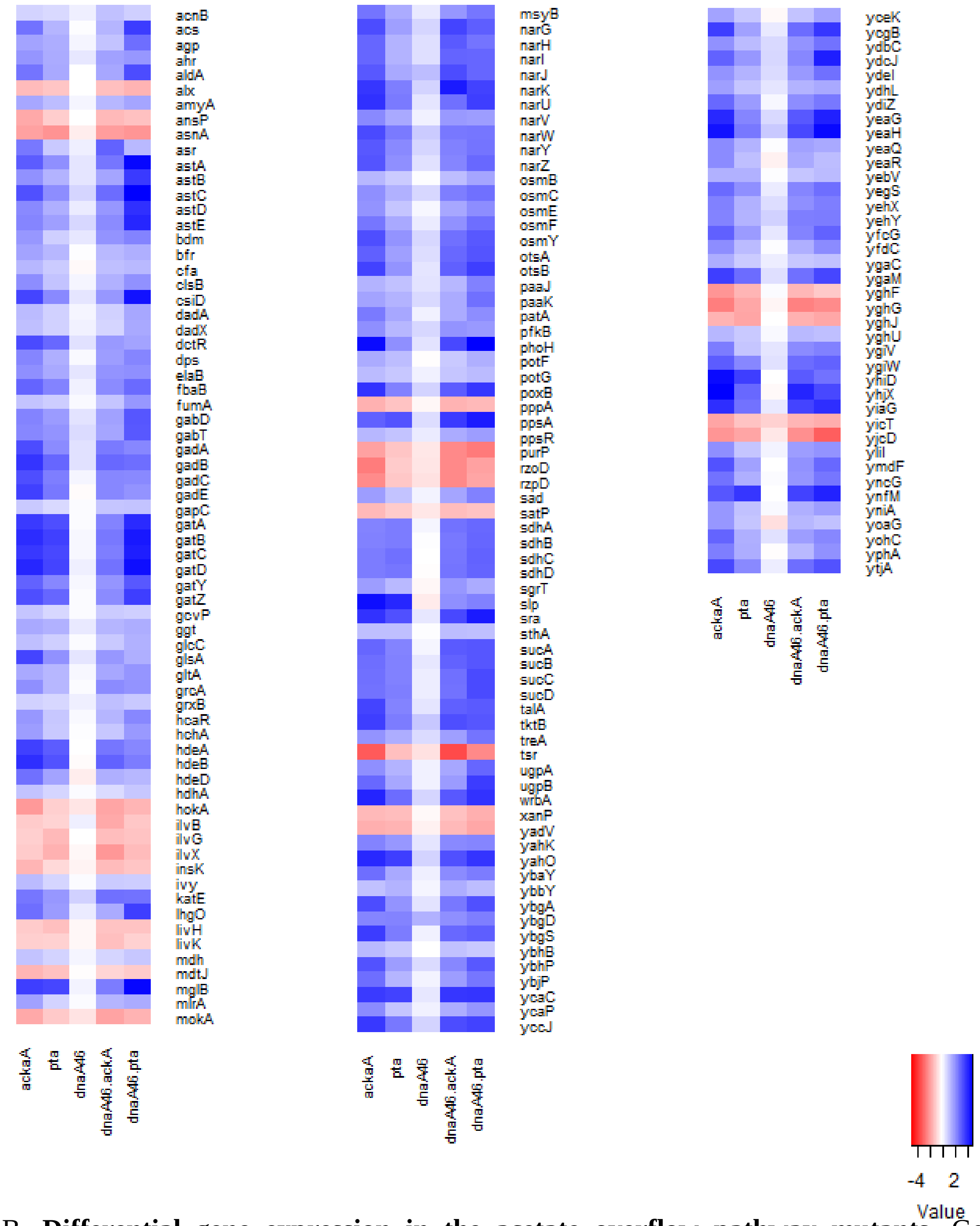

Fig B. **Differential gene expression in the acetate overflow pathway mutants.** Gene expression was estimated in the exponential phase by RNA-seq. Heat map of the genes whose expression in the metabolic mutants was changed at least **2-fold** with **p-value < 0.05**

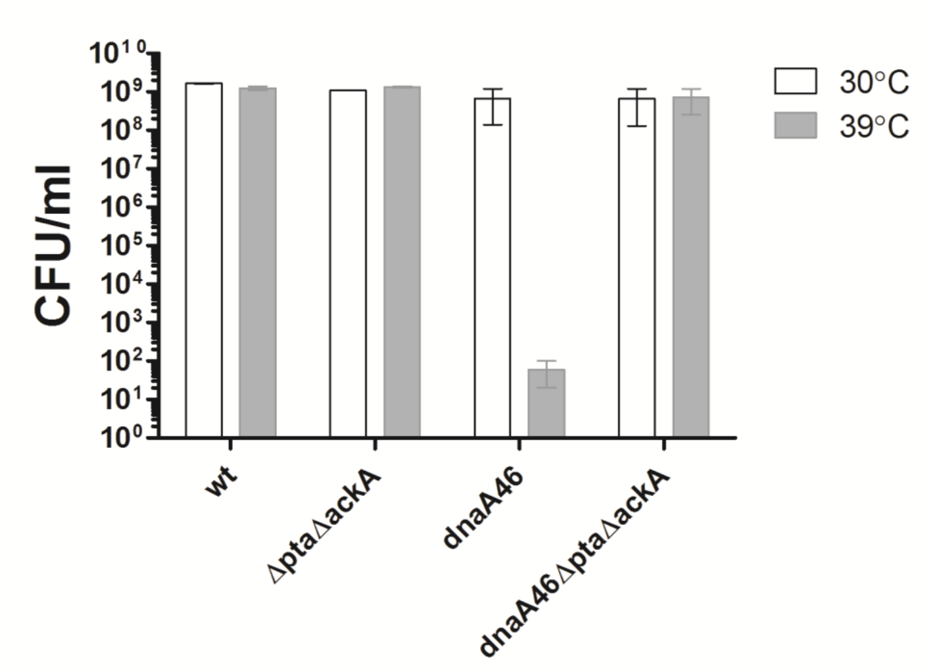

Fig C. **Suppression of temperature-sensitive growth of the *dnaA46* strain by deletion of both acetate overflow pathway genes**

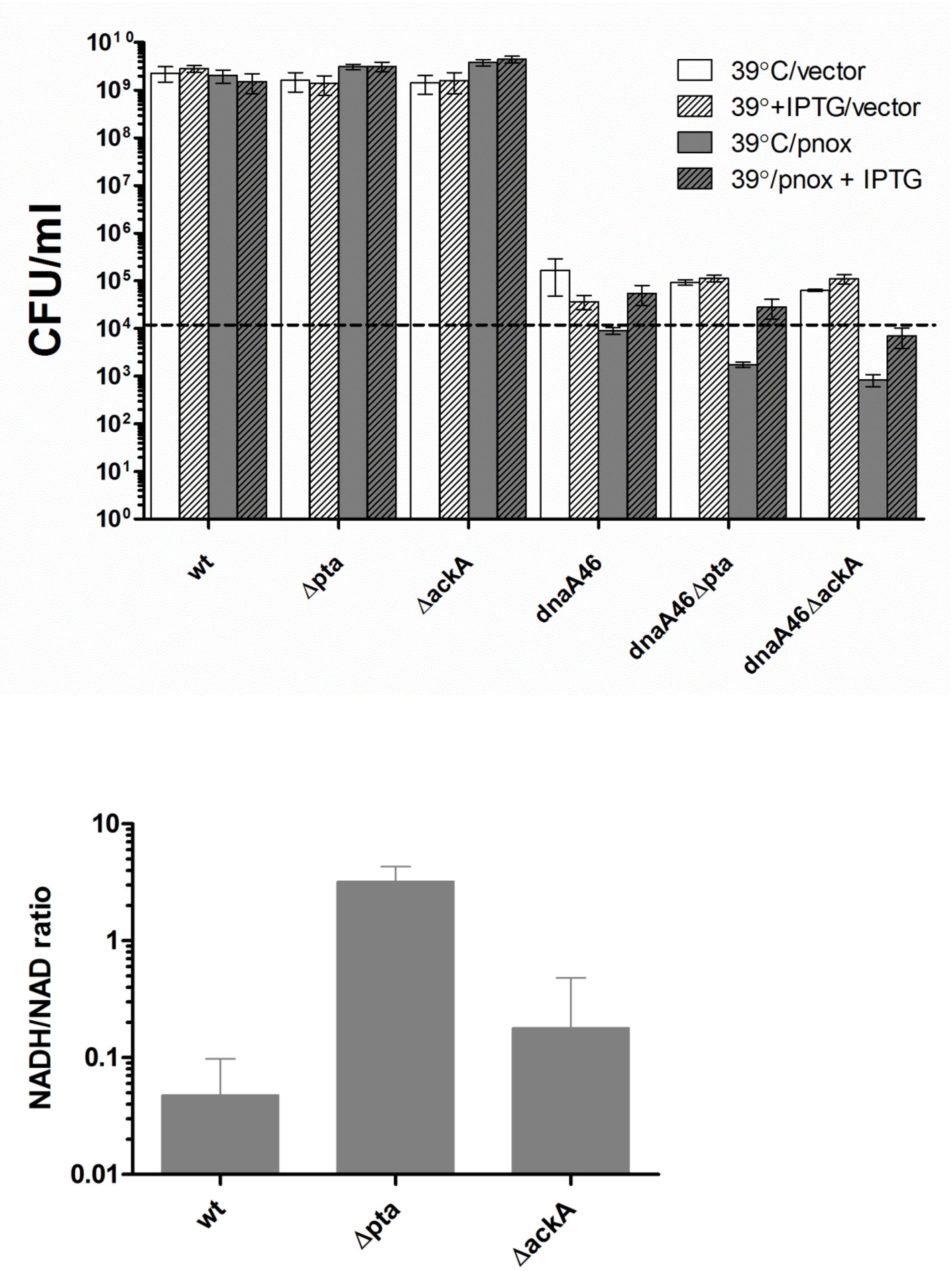

Fig D. 1) The effect of *nox* overexpression on the suppression of *dnaA46* replication defect by mutations in the acetate overflow pathway. Bacteria were grown on LB plates containing 100 mM sodium formate and 1 mM IPTG (where indicated). Growth was estimated by CFU. Data represents mean ± SEM of three independent experiments. Dashed line marks CFU value obtained for *dnaA46* strain at 39°C. **2) NADH/NAD ratio is elevated in the acetate overflow pathway mutants.** Analysis of the NADH/NAD ratio in cellular extracts was performed as described in the Materials and Methods section.

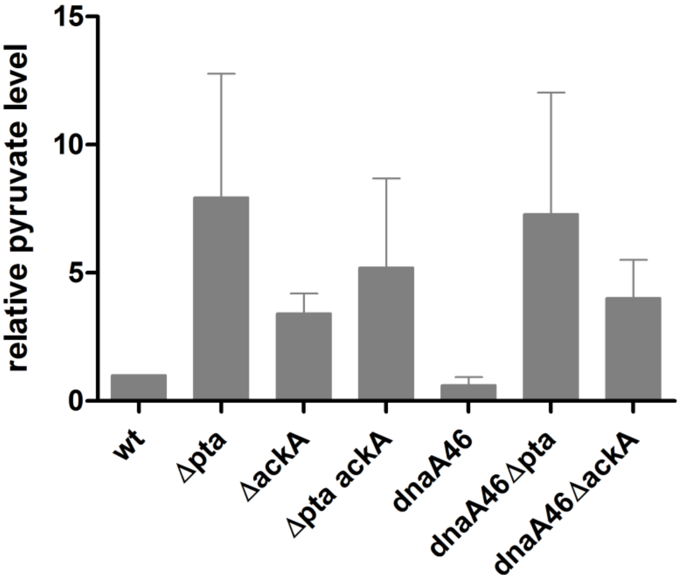

Fig E. Intracellular accumulation of pyruvate in the acetate overflow pathway mutants. Bacteria were grown at 30°C in LB medium. Concentration of pyruvate was measured as described in Materials and Methods. Data represents mean ± SEM of two independent experiments.

Table A. **Differentially expressed genes regulated by rpoS and ppGpp.** Values represent log_2 of the fold change of mRNA abundance with respect to the wild-type strain. Genes were selected whose expression in the metabolic mutants was changed **at least 2-fold with p-value < 0.05.** Values represent log2 of fold change.

		ackA	pta	dnaA 46	dnaA46 ackA	dnaA46 pta	
rpoS	acs	3.75	2.02	0.07	2.01	5.18	acetyl-CoAsynthetase
	actP	2.29	1.36	0.11	0.96	3.97	acetate transporter
	aldB	2.85	1.63	0.02	1.63	3.24	Aldehyde dehydrogenase B
	appA	1.36	0.79	0.328	1.36	1.22	phosphoanhydridephosphorylase
	appY	1.53	0.58	-0.78	1.51	1.59	Global transcriptional activator; DLP12prophage
	asr	3.59	1.47	0.44	4.18	1.89	acid shock-inducible periplasmic protein
	astB	2.98	2.05 7	0.74	2.43	5.28	Succinylarginine dihydrolase , L-arginine degradation
	astD	3.19	2.17	0.58	2.78	5.52	Succinylglutamate semialdehyde dehydrogenase , L-arginine degradation
	astE	3.28	2.59	1.09	3.11	5.79	L-arginine degradation
	cbpM	1.63	0.54	0.27	1.35	1.23	modulator of CbpA co-chaperone
	cfa	1.93	1.42	-0.17	1.73	1.91	Cyclopropane fatty acyl phospholipid synthase
	csiE	2.47	1.21	0.49	1.64	2.85	Stationaryphase inducible protein
	ddpA	2.08	1.42	0.08	1.30	3.20	D-ala-D-a la transporter subunit
	ddpB	1.56	1.15	0.40	1.19	2.57	D-ala-D-a la transporter subunit
	ddpF	1.39	0.71	0.72	1.77	1.86	D-ala-D-a la transporter subunit
	ddpX	3.06	2.29	0.48	1.54	4.14	D-ala-D-aladipeptidase
	gabP	2.59	1.68	-0.26	1.36	4.16	gamma-aminobutyrate transporter
	gabT	3.21	2.92	0.97	2.45	4.45	4-aminobutyrate aminotransferase
	gadA	4.79	3.13	0.85	3.54	3.22	Glutamate decarboxylase A
	gadB	5.40	4.08	0.85	4.04	3.85	Glutamate decarboxylase B
	gadC	4.72	3.43	0.27	3.23	3.16	glutamate:gamma-aminobutyricacidantiporter
	glgA	1.60	0.45	0.43	1.21	1.66	glycogensynthase
	glgC	1.86	0.50	0.43	1.45	1.89	glucose-1-phosphate adenylyltransferase
	glgP	1.54	0.57	0.31	1.12	1.52	glycogenphosphorylase
	glgX	1.56	0.32	0.21	1.12	1.53	glycogen debranching enzyme
	hdeA	5.07	4.36	0.02	3.67	3.23	periplasmic protein that plays a role in resistance to low pH
	hdeB	5.58	4.61	-0.14	4.18	3.52	periplasmic acid stress chaperone
	hyaA	1.71	0.65	0.66	1.49	1.28	subunit of hydrogenase 1
	hyaB	1.41	0.81	0.44	1.50	1.36	large subunit of hydrogenase 1
	katE	3.66	2.83	1.23	3.8	3.8	Catalase
	lhgO	3.87	2.69	0.56	2.27	5.17	L-2-hydroxyglutarate oxidase
	lsrA	1.48	1.13	1.05	1.12	2.24	autoinducer 2 import ATP-binding protein
	lsrB	2.20	1.20	0.65	1.18	3.31	autoinducer 2-binding protein
	mtdE	2.91	1.81	0.19	1.76	1.3	Anaerobic multidrug efflux transporter
	patD	1.54	1.31	0.33	1.10	2.35	gamma-aminobutyraldehydedehydrogenase
	pfkB	2.16	1.41	0.765	2.08	1.97	6-phosphofructokinase II
	phr	1.58	0.65	0.13	1.00	1.45	Deoxyribodipyrimidine photolyase

	poxB	3.9	2.44	0.87	3.11	3.82	Pyruvate dehydrogenase (pyruvateoxidase)
	puuB	1.96	0.60	1.05	1.79	1.92	gamma-glutamyl putrescine oxidoreductase
	puuC	2.05	0.47	1.19	2.03	2.19	gamma-glutamyl-gamma-aminobutyraldehyde dehydrogenase; succinate semialdehyde dehydrogenase
	sucA	2.94	2.39	0.20	3.15	3.23	2-oxoglutarate decarboxylase
	sucB	2.75	2.44	0.48	3.01	3.21	Dihydrolipoyl transsuccinase
	sucC	2.73	2.43	0.35	2.73	3.47	succinyl-CoAsynthetase
	sucD	2.69	2.45	0.36	2.70	3.44	succinyl-CoAsynthetase
	tktB	3.67	2.48	1.10	3.67	3.22	transketolase 2
	yehX	2.50	1.54	0.68	2.22	2.55	putative ABC superfamily transporter ATP-binding subunit
	yehY	2.45	1.52	0.91	2.51	2.55	inner membrane putative ABC superfamily transporter permease
	yhiD	4.81	3.79	-0.02	3.24	2.78	putative Mg(2+) transport ATPase
	yhjG	5.52	1.05	0.45	1.88	2.45	putative inner membrane-anchored periplasmic AsmA family protein
	yjcH	2.90	1.90	0.01	1.64	4.77	DUF485 family inner membrane protein
ppGpp	acnA	1.64	0.84	0.32	1.46	1.79	aconitatehydratase 1
	aidB	2.51	0.61	0.32	1.94	2.03	DNA alkylation damage repair protein; flavin-containing DNA binding protein
	astA	4.36	3	0.71	3.68	6.66	Arginine succinyltransferase, L-arginine degradation
	astC	4.65	3.07	1.14	3.91	6.83	Succinyl ornithine transaminase, L-arginine degradation
	blc	2.17	0.73	0.19	1.30	2.17	outer membrane lipoprotein (lipocalin)
	cbpA	1.86	0.72	0.12	1.12	1.31	DnaK co-chaperone; curved DNA-binding protein
	csiD	5.00	3.16	0.73	2.81	6.33	Carbon starvation protein
	dps	3.29	2.15	0.17	2.66	3.31	Fe-binding and storage protein; stress-inducibleDNA-binding protetin
	fbaB	4.11	3.31	0.41	3.17	3.97	fructose-bisphosphate aldolase class I
	fic	2.04	0.71	0.34	1.50	1.88	stationary-phase adenosine monophosphate-protein transferase domain protein
	gabD	3.33	2.71	0.79	2.51	4.50	succinate-semialdehyde dehydrogenase I
	gadE	5.1	3.6	-0.13	3.22	2.92	gad regulon transcriptional activator
	gadX	2.93	0.96	-0.38	1.70	2.37	Acid resistance regulon transcriptional activator; autoactivator
	glgS	2.72	0.91	-0.30	1.07	1.84	motility and biofilm regulator
	hchA	2.66	1.45	0.06	1.52	2.74	amino acid deglycase that repairs glyoxal- and methylglyoxal-glycated proteins
	mglA	3.89	3.90	-0.32	1.87	5.43	fused methyl-galactoside transporter subunits of ABC superfamily: ATP-binding components
	mglB	5.16	4.96	0.33	3.47	6.66	methyl-galactoside transporter subunit
	mglC	4.12	4.18	-0.21	2.22	5.84	methyl-galactoside transporter subunit
	mlrA	2.52	1.21	0.13	1.98	2.29	transcriptional activator of csgD and csgBA
	msyB	2.64	1.61	0.45	1.99	2.60	multicopy suppressor of secY and secA
	murQ	1.55	0.43	0.40	1.07	1.33	N-acetylmuramic acid 6-phosphate (MurNAc-6-P)etherase

	narU	3.95	2.54	0.49	2.73	3.69	nitrate/nitrite transporter
	osmB	1.38	1.02	0.06	1.30	1.73	lipoprotein
	osmC	2.17	1.49	0.78	2.50	2.74	lipoyl-dependent Cys-basedperoxidase
	osmE	2.07	1.13	0.17	0.17	2.21	osmotically-inducible lipoprotein
	osmF	2.62	1.65	0.40	2.12	2.75	putative ABC superfamily transporter periplasmic-binding protein
	osmY	3.39	2.09	0.77	2.72	3.19	periplasmic protein
	otsA	3.05	1.86	0.68	2.89	3.27	trehalose-6-phosphate synthase
	otsB	3.60	2.07	0.53	3.04	3.69	trehalose-6-phosphate phosphatase
	sra	3.91	3.34	0.47	3.48	4.36	stationary-phase-induced ribosome-associated protein
	talA	3.56	2.39	0.53	3.04	3.15	transaldolase A
	tam	1.58	0.96	0.08	1.32	1.85	trans-aconitate methyltransferase
	treA	2.06	1.57	0.71	1.85	2.66	Periplasmic trehalase
	treF	1.69	0.72	0.33	1.25	1.34	Cytoplasmic trehalase
	wrbA	4.16	2.80	0.83	3.17	3.9	NAD(P)H:quinone oxidoreductase
	ybgA	3.43	2.08	0.54	2.57	3.35	DUF1722 family protein
	ybhB	1.35	1.04	0.00	1.20	1.06	kinase inhibitor homolog
	ybjP	2.78	1.44	0.24	1.89	2.61	lipoprotein
	yccJ	3.78	2.57	0.85	3.43	3.59	uncharacterized protein
	yciF	1.69	1.28	-0.32	2.12	2.12	putative rubrerythrin/ferritin-likemetal-binding protein
	yciG	3.01	1.67	0.45	2.71	3.58	KGG family protein
	yegS	2.91	2.20	0.41	2.42	2.85	Phosphatidylglycerol kinase
	ygaU	2.49	1.16	0.38	1.81	2.31	uncharacterized protein
	yiaG	4.12	2.75	0.46	3.68	4.10	HTH_CROC1 family putative transcriptional regulator
rpoS + ppGpp	agp	2.47	2.20	0.30	1.61	3.85	glucose-1-phosphatase/inositol phosphatase
	ahr	2.87	2.29	0.69	2.52	2.81	Aldehyde reductase
	bfr	2.48	1.94	0.08	1.89	2.14	bacterioferritin
	curA	2.28	1.04	0.22	2.08	2.49	curcumin/dihydrocurcuminreductase
	dkgA	1.63	0.59	0.42	1.35	1.64	methylglyxoal reductase
	elaB	2.99	2.32	0.72	2.90	3.02	DUF883 family protein
	ggt	2.32	2.10	0.63	2.00	2.19	γ-glutamyl transpeptidase
	ldtE	1.92	0.81	0.06	1.18	1.90	Murein L
	psiF	2.41	1.17	0.14	1.24	2.31	PsiF family protein
	qorA	1.87	0.49	0.15	1.08	1.69	Quinone oxidoreductase
	rpoS	1.44	0.58	0.31	1.47	1.05	RNA polymerase sigma subnit
	slp	4.61	4.13	-0.40	2.17	2.43	Outer membranę lipoprotein
	sufA	1.71	0.57	0.39	1.52	1.85	Fe-S cluster assembly protein
	sufB	1.45	0.51	0.31	1.44	1.85	component of SufBCD Fe-S cluster assembly scaffold
	sufC	1.54	0.63	0.45	1.56	1.63	SufBCD Fe-S cluster assembly scaffold protein
	sufD	1.43	0.65	0.49	1.48	1.63	component of SufBCD Fe-S cluster assembly scaffold
	ugpB	2.89	1.68	0.27	1.94	3.73	glycerol-3-phosphate transporter subunit
	yahK	2.40	2.01	0.53	2.27	2.36	Aldehyde reductase
	yahO	4.10	3.68	0.85	3.33	3.88	Periplasmic protein

	ybaT	3.81	2.43	0.05	2.07	1.53	Putative aminoacid transporter
	ybaY	2.78	1.67	0.32	2.22	2.51	Outer membranę lipoprotein
	ybdK	2.86	1.31	0.45	2.35	3.11	Weak gamma-glutamyl:cysteineligase
	ybeL	2.85	1.23	0.28	1.79	2.76	DUF1451 family protein
	ybhP	3.32	1.88	0.72	1.19	1.06	kinase inhibitor homolog
	ycaC	3.75	3.60	0.48	3.84	3.89	Putative isochorismatase family hydrolase
	ycaP	2.25	1.20	0.28	1.57	2.06	UPF0702 family putative inner membrane protein
	ycgB	3.78	1.87	0.45	2.87	3.95	SpoVR family stationary phase protein
	ydcK	2.24	0.99	0.37	1.85	2.45	uncharacterized protein
	ydeI	2.14	1.53	0.74	2.02	2.83	hydrogen peroxide resistance OB fold protein;putativeperiplasmic protein
	yeaG	4.21	2.40	0.78	3.30	4.40	protein kinase
	yeaH	4.65	2.66	1.07	3.57	4.84	UPF0229 family protein
	yeaQ	2.30	1.52	0.05	1.86	1.70	UPF0410 family protein
	yebV	1.56	1.55	0.04	1.16	1.37	uncharacterized protein
	yedP	2.48	1.03	0.08	1.67	2.33	putative mannosyl-3-phosphoglyceratephosphatase
	yegP	2.60	1.23	-0.18	1.04	2.22	UPF0339 family protein
	yfcG	3.12	1.96	0.48	2.45	3.07	GSH-dependent disulfide bond oxidoreductase
	ygaM	3.77	2.87	0.66	2.80	3.63	DUF883 family protein
	yghA	2.30	1.00	0.34	2.11	2.47	Putative oxidoreductase
	yhbO	1.26	0.12	0.16	1.06	1.25	stress-resistance protein
	yhcO	3.05	1.30	0.53	2.45	2.94	Putative barnase inhibitor
	yhfG	1.47	0.68	-0.01	1.06	1.36	putative Fic-binding protein
	yhhA	2.03	0.83	0.04	1.39	1.99	DUF2756 family protein
	yjbJ	2.52	1.10	0.35	1.57	2.50	stress-induced protein
	yjdJ	2.74	0.69	0.12	1.99	2.29	putative acyl-CoA transferase
	yjdN	2.86	1.13	0.54	1.59	2.56	Metalloprotein superfamily protein
	yodD	1.82	0.79	-0.11	1.02	1.78	uncharacterized protein
	yohC	3.06	1.60	0.63	1.94	2.39	Yip1 family inner membrane protein
	ysgA	2.46	0.95	0.41	1.50	2.43	Putative carboxymethylene butenolidase

Table B. **Expression of genes encoding transport proteins.** Differences between wild-type and mutant strains are depicted as fold change.

gene name	Relative mRNA level (FC)			
	pta	*ackA*	*dnaA46pta*	*dnaA46ackA*
mglA	14.9	14.8	42.8	3.6
mglB	31.1	35.5	101.1	11.1
mglC	18.1	17.4	57.3	4.66
gatY	9.2	18	21.8	7.2
gatZ	16.8	26.2	35	74.5
malE	2.9	29.8	183	2.7
malF	2	30.3	136.2	3.1
malG	1.4	14.7	83.9	1.88
malK	2.7	89.3	458	6.3
lamB	3.4	49.9	439.5	3.2
